# Seroprevalence of *Toxoplasma gondii* in pregnant women and livestock in the mainland of China: a systematic review and hierarchical meta-analysis

**DOI:** 10.1038/s41598-018-24361-8

**Published:** 2018-04-18

**Authors:** Huifang Deng, Brecht Devleesschauwer, Mingyuan Liu, Jianhua Li, Yongning Wu, Joke W. B. van der Giessen, Marieke Opsteegh

**Affiliations:** 1 Centre for Infectious Disease Control - Zoonoses and Environmental Microbiology, National Institute for Public Health and the Environment, 3720 BA Bilthoven, Netherlands; 2 Department of Public Health and Surveillance, Scientific Institute of Public Health (WIV-ISP), 1050 Brussels, Belgium; 3 Institute of Zoonosis, Jilin University, 130062 Changchun, People’s Republic of China; 4 Key Laboratory of China Food Safety Risk Assessment, National Center for Food Safety Risk Assessment, 100022 Beijing, People’s Republic of China

## Abstract

Primary *Toxoplasma gondii* infection in pregnant women may result in abortion, stillbirth, or lifelong disabilities of the unborn child. One of the main transmission routes to humans is consumption of raw or undercooked meat containing *T. gondii* tissue cysts. We aim to determine and compare the regional distribution of *T. gondii* seroprevalence in pregnant women and meat-producing livestock in China through a systematic literature review. A total of 272 eligible publications were identified from Medline, Scopus, Embase and China National Knowledge Infrastructure. Apparent and true seroprevalence were analysed by region using a novel Bayesian hierarchical model that allowed incorporating sensitivity and specificity of the applied serological assays. The true seroprevalence of *T. gondii* in pregnant women was 5.0% or less in seven regions of China. The median of the regional true seroprevalences in pigs (24%) was significantly higher than in cattle (9.5%), but it was not significantly higher than in chickens (20%) and small ruminants (20%). This study represents the first use of a Bayesian hierarchical model to obtain regional true seroprevalence. These results, in combination with meat consumption data, can be used to better understand the contribution of meat-producing animals to human *T. gondii* infection in China.

## Introduction

*Toxoplasma gondii* is an obligate intracellular protozoan parasite that causes toxoplasmosis. The parasite is widely distributed in the world and can infect a wide range of warm-blooded animals, including humans, pets and livestock. In the general population, *T. gondii* infection can remain asymptomatic, cause lymphadenopathy and flu-like symptoms, or lead to eye disease, most frequently chorioretinitis, while in immune-compromised patients, it can be fatal^1^. Pregnant women constitute a specific risk group: if primary infection is acquired during pregnancy, this may lead to abortion, stillbirth and neurological disorders in the unborn child^2^. Congenital and acquired toxoplasmosis caused more than 20 million new cases worldwide in 2010, resulting in an estimated global disease burden of 1.68 million (95% UI 1.24–2.45 million) disability-adjusted life years (DALYs), of which 829,000 DALYs (95% UI 561,000–1.26 million) were estimated to be foodborne^3^. In a global multicriteria based ranking (considering public health, animal health, microbial ecology, agribusiness and trade, and socio-economic impact) *T. gondii* ranked fourth out of 24 foodborne parasites^4^.

The main routes of postnatal infection for humans are consumption of raw or undercooked meat containing tissue cysts and food or water contaminated with sporulated oocysts shed by the primary infected definite hosts, felines^1^. As intermediate hosts of *T. gondii*, meat-producing animals serve as one of the main sources of human infections^5^. It was estimated that 30–63% of infections in pregnant women from six large European cities was attributed to meat^6^. As the effectiveness of treatment is unclear^7,8^, prevention of infection is so far the most important strategy but relies on knowledge of the relative attribution of different transmission routes. Results from an extensive literature review showed that there is a positive relationship between detection of antibodies to *T. gondii* and presence of this parasite in pigs, chickens and small ruminants, but not in cattle and horses^9^. Thus, with the exception of cattle and horses the seroprevalence and geographical distribution of the *T. gondii* infection in different meat-producing animals gives an indication of the risk of human infection via consumption of undercooked meat and is useful for developing health education material for pregnant women and other risk groups. In China, the seroprevalence of *T. gondii* in two national surveys conducted in 1988–1992 and 2001–2004 has increased from 5.2% to 7.9%^10^. The geographical distribution of the *T. gondii* seroprevalence in the general population and meat-producing animals is not reported systematically and most of the studies were published in Chinese which are not easily accessible for the international scientific community. Apart from the two national surveys, studies concerning *T. gondii* infection in the general population were hardly available, thus we decided to use data from pregnant women as a proxy for the general population.

In epidemiological studies true prevalence (TP) rather than apparent prevalence (AP) is the parameter of interest, and requires information on test sensitivity (Se) and specificity (Sp). A variety of serological assays have been developed for the detection of *T. gondii-*specific immunoglobulins^11^ and enzyme-linked immunosorbent assay (ELISA), modified agglutination test (MAT), and indirect hemagglutination antibody test (IHA) are commonly used in China. Unfortunately, estimates of Se and Sp are often lacking or evaluated in the absence of appropriate reference tests or on samples that are not relevant for the target population. Bayesian modelling is therefore increasingly used for veterinary epidemiological studies to infer true prevalence while taking into account the uncertainty of Se and Sp^12–16^. Furthermore, the Bayesian framework allows explicitly modelling complex hierarchical structures, such as studies nested within regions, which in turn are nested within a country. This has the advantageous side-effect that regions in which few or no studies were performed, can “borrow strength” from the remaining regions, and data gaps can be imputed^14,17^. To our knowledge, however, both applications i.e., true prevalence estimation and hierarchical modelling with data imputation has never been combined in a single model.

The present study estimates the seroprevalence of *T. gondii* in pregnant women and in the main meat-producing animals (i.e., pig, cattle, sheep, goat, chicken, duck, goose and donkey) from different regions of China and analyses the potential link between the seroprevalence in humans and livestock. Data on apparent seroprevalence were collected by systematically reviewing international and Chinese bibliographic databases. A Bayesian hierarchical model that allowed incorporating the sensitivity and specificity of the applied serological assays was used to estimate true prevalence by region and impute possible data gaps.

## Results

### Characteristics of eligible studies

**Figure 1:**
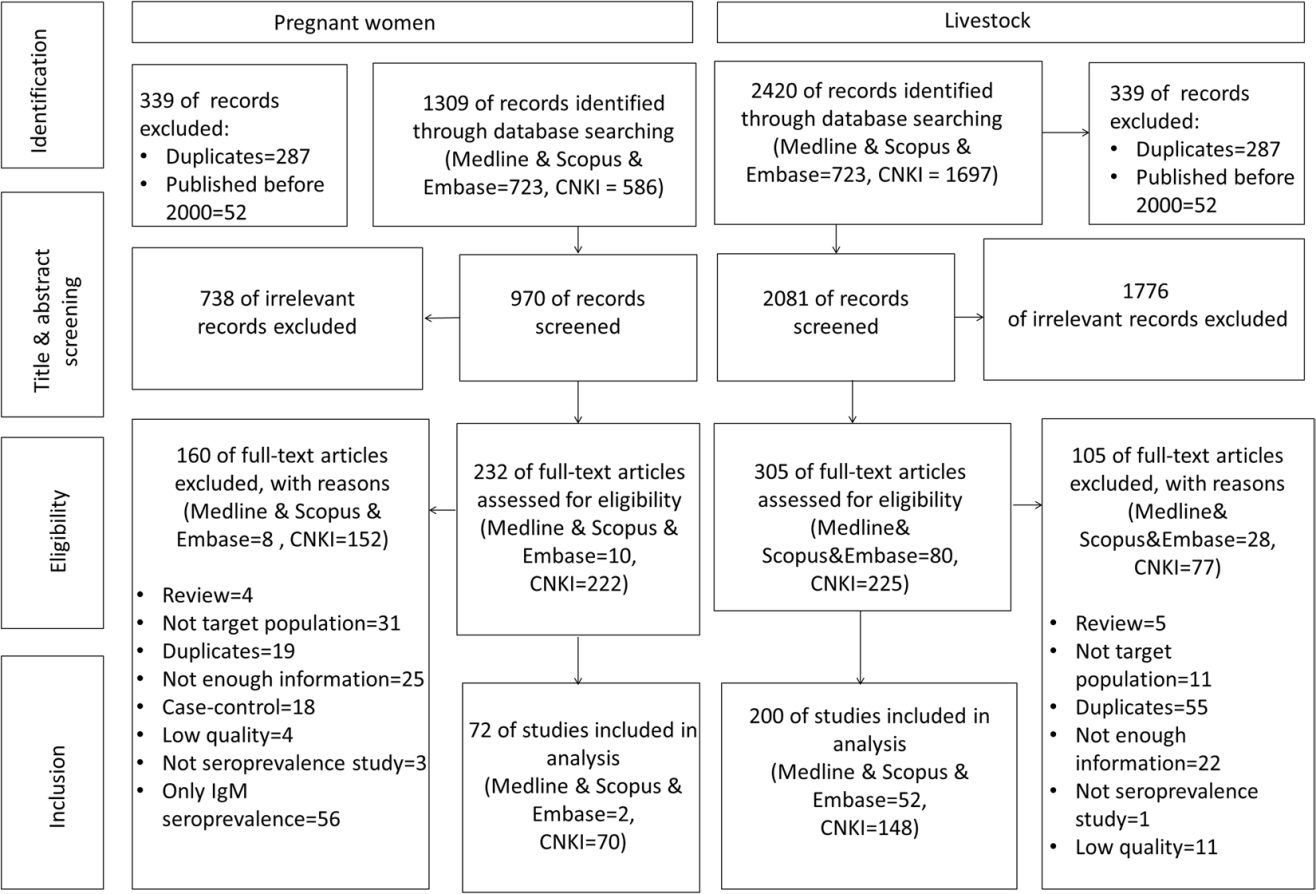
Flowchart: search strategy steps and selection of relevant studies on *T. gondii* seroprevalence in pregnant women and livestock in mainland of China. Five steps were included to select relevant studies for pregnant women and livestock in mainland of China.

The selection process of published papers for pregnant women and livestock is summarized in a Preferred Reporting Items for Systematic Reviews and Meta-Analyses (PRISMA) flow diagram as shown in Fig. 1. A total of 72 eligible studies which reported the seroprevalence of IgG antibodies to *T. gondii* for pregnant women were collected from 21 provinces of China; no eligible studies were conducted in Chongqing, Fujian, Hainan, Jiangxi, Neimenggu, Ningxia, Qinghai, Tianjin, Yunnan provinces or the Tibet Autonomous Region (Xizang). The included pregnant women were at different weeks of their pregnancy, and the information of mean age was available from 31 studies, and ranged from 25 to 32 years old.

**Figure 2:**
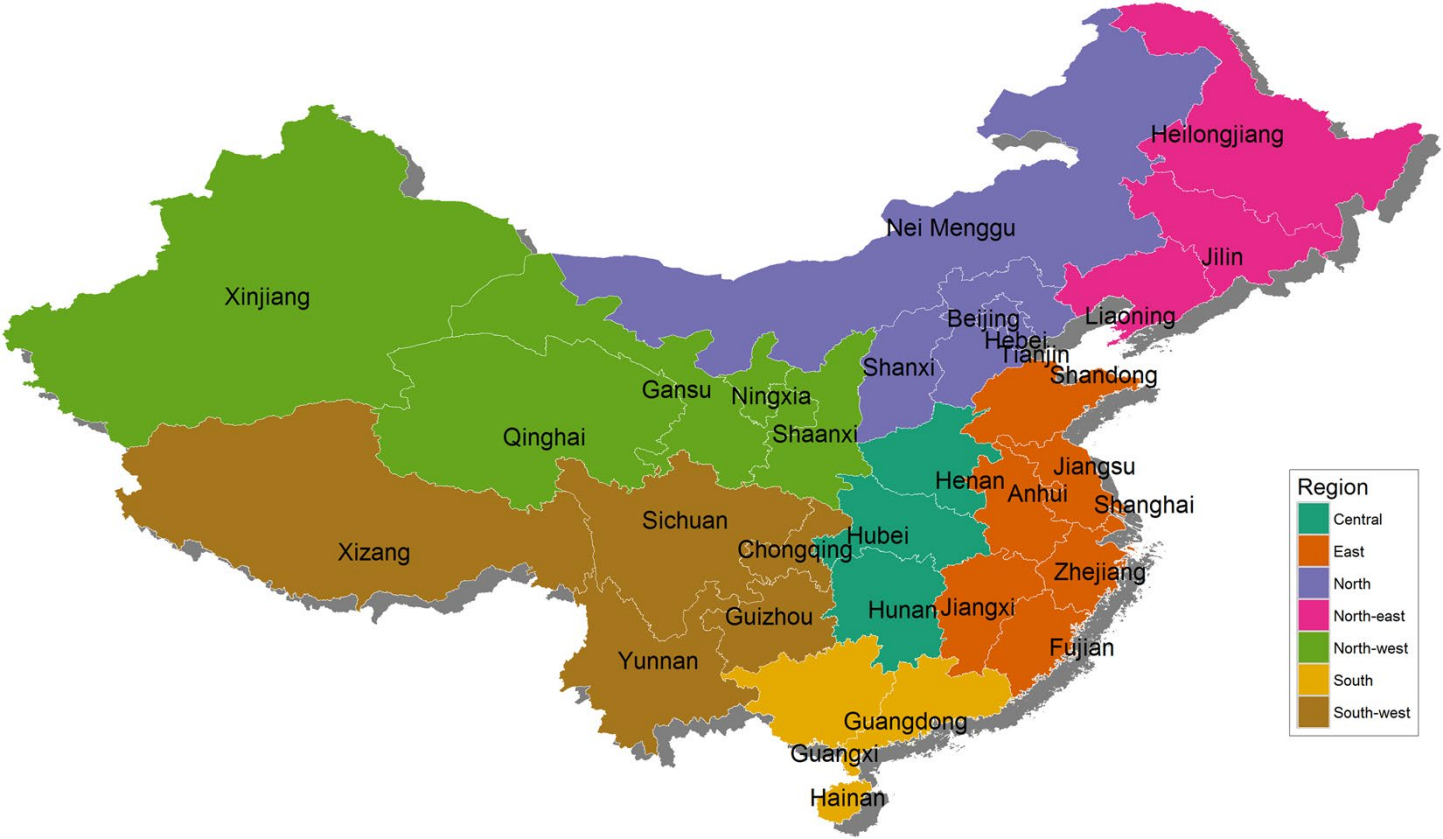
Seven regions from 31 provinces of mainland China. Thirty-one provincial-level divisions of mainland China were grouped as Central, East, North, Northeast, Northwest, South and Southwest China. Geographic data for mainland China was obtained from “Global Administrative Areas” (http://www.gadm.org/home). Map was created using R version 3.3.1 (https://www.r-project.org/).

The literature review identified 200 publications for livestock from 30 provinces. None of the included studies were performed in Tianjin province. A total number of 240 studies were published in these 200 publications. The studies for pregnant women from 21 provinces and for pigs, chickens, cattle and small ruminants from the 30 provinces were merged to seven regions of China (Fig. 2). The entire list of 272 selected studies can be found as Supplementary File S1.

### Seroprevalence of pregnant women

In pregnant women, a small variation of non-modelled apparent prevalence (obtained by summing the data per region) was observed between different regions of China. The non-modelled AP ranged from 2.1% to 11% in all seven regions (Table 1). The apparent seroprevalence estimates from the hierarchical model were similar to the results of non-modelled AP. The highest mean AP from the hierarchical model was identified in Northeast (5.7%, 95% PPI (posterior prediction interval) [2.9–11%]), and the lowest in Northwest China (3.1%, 95% PPI [1.2–5.4%]) (Table 2).

**Table 1:** Non-modelled apparent seroprevalence (%) of pregnant women and livestock in mainland of China.

	Pig		Chicken		Small ruminant		Cattle		Pregnant women	
	AP (%) (95% CI) (Pos./Tot.)	No.	AP (%) (95% CI) (Pos./Tot.)	No.	AP (%) (95% CI) (Pos./Tot.)	No.	AP (%) (95% CI) (Pos./Tot.)	No.	AP (%) (95% CI) (Pos./Tot.)	No.
Central	24 (23–24) (4711/19823)	16	17 (15–18) (348/2082)	6	11 (10–12) (592/5409)	6	16 (13–20) (66/405)	3	6.8 (6.6–7.0) (5672/83379)	13
East	24 (23–24) (7568/31627)	29	22 (20–24) (348/1614)	8	17 (15–19) (179/1066)	6	7.0 (3.9–12) (14/199)	5	4.6 (4.5–4.7) (4449/96953)	28
North	26 (24–28) (647/2470)	4	38 (33–42) (179/474)	2	14 (12–16) (239/1706)	4	18 (12–26) (23/128)	3	5.5 (5.1–5.8) (1131/20743)	10
Northeast	12 (11–13) (656/5476)	4	11 (9.8–12) (428/3995)	8	6.5 (5.6–7.4) (196/3020)	4	6.9 (6.1–7.8) (240/3478)	6	8.9 (8.1–9.8) (400/4480)	7
Northwest	27 (26–29) (1616/5905)	17	7.7 (5.9–9.9) (56/729)	5	15 (14–15) (1937/13077)	27	16 (16–17) (1637/10009)	19	2.1 (1.9–2.4) (316/14831)	6
South	18 (17–20) (419/2296)	3	17 (15–19) (218/1280)	5	/	0	/	0	6.1 (5.5–6.8) (363/5928)	6
Southwest	53 (52–54) (13550/25536)	23	31 (24–38) (54/177)	2	18 (17–19) (838/4711)	7	5.8 (3.8–8.5) (25/430)	3	11 (9.1–12) (158/1489)	2

AP: apparent prevalence; Pos.: number of seropositive animals; Tot.: number of tested animals; No.: number of records.

**Table 2:** Hierarchical model estimates for *T. gondii* apparent seroprevalence (%) in pregnant women and livestock in mainland of China, by region.

	Pig	Chicken	Small ruminant	Cattle	Pregnant women
	AP (%) (95%PPI)	AP (%) (95%PPI)	AP (%) (95%PPI)	AP (%) (95%PPI)	AP (%) (95%PPI)
Central	22 (15–31)	13 (6.5–22)	8.8 (4.6–14)	8.9 (3.7–17)	5.1 (3.0–8.7)
East	20 (14–27)	19 (10–31)	12 (6.9–23)	7.1 (2.3–13)	3.2 (2.1–4.5)
North	21 (10–33)	21 (8.8–44)	11 (6.1–21)	11 (5.2–25)	4.1 (2.3–6.8)
Northeast	18 (7.7–29)	10 (5.0–17)	8.6 (4.0–15)	7.7 (3.5–13)	5.7 (2.9–11)
Northwest	18 (11–26)	10 (3.9–18)	8.4 (5.7–12)	10 (6.4–15)	3.1 (1.2–5.4)
South	20 (9.2–34)	16 (8.2–28)	11 (3.6–25)	9.6 (2.2–25)	3.6 (1.7–6.3)
Southwest	29 (20–41)	20 (8.5–43)	11 (6.4–19)	8.4 (3.2–16)	5.6 (2.4–14)

AP: apparent seroprevalence; PPI: posterior probability intervals.

Extracted information of Se and Sp from the commercial kits used in pregnant women in the selected studies and the parameters of beta distribution used in the Bayesian hierarchical model are shown in Table 3. A total number of 49 out of 72 studies used commercial kits with unknown test characteristics. The mean posterior seroprevalences in Chinese pregnant women and the mean posterior estimates for serological test characteristics are shown in Tables 4 and 5. The Se and Sp adjusted seroprevalences in Chinese pregnant women ranged from 2.4% to 5.0% among different regions. Trace plots and the potential scale reduction factors showed good convergences for the parameters in all models. The codes for the Bayesian hierarchical model can be found as Supplementary File S2.

**Table 3:** Reported sensitivities and specificities of commercial serological kits for detection of anti-*T. gondii* antibodies used in records included for pregnant women and livestock and the prior distributions used in the Bayesian hierarchical model.

	Kit	Method	Se (%)	Distribution Se	Sp (%)	Distribution Sp	No. of records	Ref
**Pregnant women**	Kit1	ELISA	100% (111/111)	Beta (112, 1) T (0.1)	99% (261/265)	Beta (261, 4) T (0.1)	1	^42^
	Kit2	ELISA	100% (16/16)	Beta (17, 1) T (0.1)	97% (72/74)	Beta (72, 2) T (0.1)	2	^43^
	Kit3	ELISA	100% (16/16)	Beta (17, 1) T (0.1)	100% (74/74)	Beta (75, 1) T (0.1)	2	^43^
	Kit4	ELISA	100% (111/111)	Beta (112, 1) T (0.1)	100% (265/265)	Beta (266, 1) T (0.1)	1	^42^
	Kit5	ELISA	88% (38/43)	Beta (38, 5) T (0.1)	100% (63/63)	Beta (64, 1) T (0.1)	3	^44,45^
	Kit6	ELISA	100% (16/16)	Beta (34, 1) T (0.1)	92% (68/74)	Beta (36, 1.5) T (0.1)	11	^43^
			93% (26/28)		97% (253/260)			^46^
			100% (18/18)		100% (392/393)			^47^
			96% (27/28)		91% (51/56)			^48^
	Kit7	ELISA	100% (16/16)	Beta (6.9, 1) T (0.5)	20% (15/74)	Beta (1.5, 1.8) T (0.5)	1	^43^
			95% (18/19)		70% (16/23)			^48^
	Kit8	ELISA	32% (9/28)	Beta (2, 2) T (0.1)	97% (61/63)	Beta (7.9, 0.9) T (0.1)	1	^49^
			69% (11/16)		87% (20/23)			^48^
	Kit9	IHA	90% (167/186)	Beta (167, 19) T (0.1)	97% (114/118)	Beta (114, 4) T (0.1)	1	^50^
	Unknown foreign Kit10			Beta (6.4, 1) T (0.1)		Beta (20, 1.1) T (0.1)	7	
	Unknown Chinese Kit11			Beta (24, 2) T (0.1)		Beta (24, 2.4) T (0.1)	43	
**Livestock**	Kit1	IHA	73% (11/15)	Beta (2.8, 1.9) T (0.1)	77% (13/17)	Beta (4.7, 0.8) T (0.1)	116	^51^
			50% (10/20)		97% (58/60)			^52^
	Kit2	ELISA	60% (12/20)	Beta (12, 8) T (0.5)	85% (51/60)	Beta (51, 9) T (0.1)	9	^52^
	Kit3	ELISA	93% (14/15)	Beta (6.5, 1.6) T (0.1)	71% (12/17)	Beta (4.4, 1.0) T (0.1)	8	^51^
			90% (18/20)		95% (57/60)			^52^
	Unknown Kit4	MAT	60% (12/20)	Beta (12, 8) T (0.1)	88% (53/60)	Beta (53, 7) T (0.1)	26	^52^
	Unknown Kit5	ELISA	/	Beta (6.9, 1.6) T (0.5)	/	Beta (10, 1.9) T (0.1)	48	
	Unknown Kit6	IHA	/	Beta (5.1, 3.2) T (0.1)	/	Beta (5.7, 0.9) T (0.1)	33

**Table 4:** Bayesian hierarchical model estimates for *T. gondii* true seroprevalence (%) in pregnant women and livestock in mainland China, by region.

	Pig	Chicken	Small ruminant	Cattle	Pregnant women
	TP (%) (95%PPI)	TP (%) (95%PPI)	TP (%) (95%PPI)	TP (%) (95%PPI)	TP (%) (95%PPI)
Central	26 (16–38)	19 (6.8–40)	18 (4.6–42)	9.5 (2.0–26)	4.3 (2.2–7.8)
East	24 (16–33)	22 (8.8–45)	24 (7.5–60)	7.1 (1.1–18)	2.4 (1.3–3.8)
North	25 (11–42)	24 (7.1–59)	21 (5.3–54)	12 (2.9–37)	3.6 (1.7–6.6)
Northeast	20 (5.8–33)	11 (3.0–28)	15 (1.9–36)	6.8 (1.3–16)	5.0 (2.2–11)
Northwest	22 (12–33)	13 (3.0–28)	16 (5.2–33)	10 (3.5–21)	2.7 (0.9–5.1)
South	24 (9.7–40)	20 (6.8–41)	20 (2.8–61)	9.8 (1.1–33)	3.0 (1.1–5.8)
Southwest	33 (22–48)	23 (7.0–56)	23 (6.8–57)	8.2 (1.4–21)	4.8 (1.7–14)

TP: true seroprevalence; PPI: posterior probability intervals.

**Table 5:** Bayesian hierarchical model estimates for sensitivities and specificities of commercial serological kits for detection of anti-*T. gondii* antibodies in pregnant women and livestock.

Population	Kit	Company	Se (%) (95% PPI)	Sp (%) (95% PPI)
**Pregnant women**	Kit 1	Abbott Laboratories	99% (97–100)	99% (98–100)
	Kit 2	BioCheck, Inc, USA	95% (80–100)	97% (96–99)
	Kit 3	CanAg	94% (80–100)	99% (97–100)
	Kit 4	Diasorin	99% (97–100)	100% (100–100)
	Kit 5	Trinity Biotech	88% (77–96)	100% (99–100)
	Kit 6	Zhuhai Haitai Biological Pharmaceuticals Company	97% (90–100)	99% (98–100)
	Kit 7	Shenzhen Jingmei Biology Engineering Company	95% (71–100)	97% (96–99)
	Kit 8	Huamei Biology Engineering Company	33% (11–81)	100% (100–100)
	Kit 9	Lanzhou Veterinary Research Institute	90% (85–94)	96% (91–99)
	Unknown foreign Kit 10	/	89% (66–100)	99% (98–100)
	Unknown Chinese Kit 11	/	93% (80–99)	100% (99–100)
**Livestock**	Kit 1	Lanzhou Veterinary Research Institute	47% (24–85)	99% (98–100)
	Kit 2	Zhuhai Haitai Biological Pharmaceuticals Company	63% (51–80)	85% (75–93)
	Kit 3	Shenzhen Combined Biotech Company	88% (59–100)	82% (44–100)
	Unknown Kit 4	/	51% (30–74)	95% (92–97)
	Unknown Kit 5	/	81% (51–98)	96% (91–99)
	Unknown Kit 6	/	56% (23–87)	99% (98–100)

### Seroprevalence of livestock

A wide variation of non-modelled apparent seroprevalence was observed in different animal species (Table 1). The non-modelled AP of *T. gondii* in ducks, goose and donkeys ranged from 9.4–27%, 1.7–21% and 0–24% respectively. However, since only few studies were eligible from ducks (5), geese (5), and donkeys (5) through the literature review, these animal species were not included in meta-analysis and correlation analysis with pregnant women. Among the other four animal species, the non-modelled AP of *T. gondii* infection was higher in pigs and chickens than in small ruminants and cattle (Table 1). The highest non-modelled AP was found in pigs (53%) in Southwest China and the lowest in cattle (5.8%) in Southwest China. No eligible studies in cattle and small ruminants were conducted in the Southern region of China.

The results of *T. gondii* apparent seroprevalence in different animals from the hierarchical models were similar to the results of non-modelled AP and they are shown in Table 2. The *T. gondii* modelled AP in small ruminants and cattle in South China were imputed as 9.6% (95% PPI, 2.2–25%) and 11% (95% PPI, 3.6–25%).

Extracted information of Se and Sp from the commercial kits used in livestock in the selected studies and the parameters of beta distribution used in the Bayesian hierarchical model are shown in Table 3. Among the 240 records, the numbers of records using kit 1–4 were 116, 9, 8 and 26 respectively, 48 studies used ELISA kits with unknown Se and Sp, 33 studies used IHA kits with unknown Se and Sp. The mean posterior estimates of the regional true seroprevalence in pigs (20–33%), chickens (11–24%), small ruminants (15–24%) and cattle (6.8–12%) are summarized in Table 4. Wide posterior probability intervals for the sensitivities and specificities were found in some of the included kits used for animals (Table 5). The Kruskal-Wallis test indicated that the median of the regional true seroprevalences in different species was significantly different from each other (*p* < 0.01). The post-hoc analysis results showed that the median of the regional true seroprevalences in pigs (24%) was significantly higher than in cattle (9.5%) (*p* < 0.05) and it was not significantly higher than in chickens (20%) and small ruminants (20%). Trace plots and the potential scale reduction factors showed good convergences for the parameters in all models.

The results of Spearman’s rank correlation coefficient showed that there is no strong relationship between the estimated true seroprevalence in pregnant women and the estimated true seroprevalence in any of the livestock species from the corresponding seven regions of China (Table 6). However, the true seroprevalence in chicken was found strongly correlated with the true seroprevalence in small ruminant (Spearman correlation = 0.86, *p* = 0.01).

**Table 6:** Correlation of regional *T. gondii* true seroprevalence between pregnant women and livestock in mainland of China.

		Pig	Chicken	Small ruminant	Cattle	Pregnant women
Pig	Spearman’s ρ	1				
Chicken	Spearman’s ρ	0.70	1			
	*p*-value	0.08				
Small ruminant	Spearman’s ρ	0.59	0.86	1		
	*p*-value	0.16	**0.01**			
Cattle	Spearman’s ρ	0.20	0.36	0	1	
	*p*-value	0.67	0.43	1		
Pregnant women	Spearman’s ρ	0.18	−0.18	−0.39	−0.35	1
	*p*-value	0.70	0.70	0.38	0.43	

## Discussion

*T. gondii* causes a high disease burden in humans and is a source of economic losses to livestock industries^18,19^. The increased seroprevalence in *T. gondii* in the Chinese human population together with a notable increase in number of immune-compromised patients suffering cancer and HIV make this opportunistic parasitic disease an important public health challenge to China^10,20^. The aims of our study were to determine the seroprevalence of *T. gondii* in pregnant women and in main meat-producing animals from different regions of China and to examine the potential link between the seroprevalence in humans and livestock.

A total of 72 publications on pregnant women and 200 publications on livestock were included in the dataset. The selected eligible studies for pregnant women covered all seven regions of China. However, data gaps were identified for cattle and small ruminant from Southern China and limited eligible studies were found in ducks, goose and donkeys. These data gaps called for the use of a hierarchical model in which the fitted national (logit-transformed) seroprevalence and the fitted between-region variance could be used to estimate the seroprevalence for regions where no data were available. In all of the eligible publications, seroprevalence data were reported without correction for test sensitivity and specificity. Correctly determining disease status largely depends on the sensitivity and specificity of the serological test. The chance of having a false result can be considerable and the prevalence of test positives may deviate from prevalence of truly infected individuals^21^. For that reason, data on test characteristics were additionally collected from literature. In our study, most of the selected studies dealing with the *T. gondii* IgG seroprevalence in pregnant women and many of the studies on livestock used kits with unknown Se and Sp. Moreover, different values of Se and Sp were reported in literature for the same kit to detect *T. gondii* antibodies in animals. This variation may be due to different characteristics of the reference population (e.g. different levels of exposure to additional pathogens or other biological confounders) and the sampling strategies used in the validation procedures^22^. Thus, it is inappropriate to assume that the test characteristics are constant over populations^23^. Therefore a Bayesian approach to estimate the true seroprevalence of *T. gondii* infection was used. In this approach, prior information about test characteristics was incorporated into the analysis as random variables described using probability distributions in the modelling process. They are therefore not fixed, constant values over different populations. Because insufficient convergence occurred from the initial runs of the model, we used truncated beta distributions for the priors of Se and Sp (Table 3) assuming that the values of Se and Sp of used kits were at least larger than 0.1. After truncation of the distribution of priors, the models for both pregnant women and livestock showed good convergence. In general, the Bayesian hierarchical model estimates for *T. gondii* true seroprevalence in pregnant women and livestock were similar to the non-modelled apparent seroprevalence but with wider 95% posterior probability intervals. In our opinion, the seroprevalences based on the Bayesian hierarchical model provide the most appropriate estimates of seroprevalence, as regional variation and information on test characteristics are taken into account. The uncertainty present due to the use of insufficiently validated diagnostic kits remains unnoticed when apparent prevalences are reported, but is reflected in the wide posterior probability intervals from the Bayesian hierarchical model. Moreover, the Bayesian hierarchical model allowed us to impute seroprevalence estimates for regions that lacked data.

The true seroprevalence from the Bayesian hierarchical model in Chinese pregnant women obtained from this study ranged from 2.4% to 5.0% in all seven regions of China. Even though the results were low compared to some countries^24^, this also means that the majority of pregnant women in China are susceptible to a primary infection and their babies to congenital toxoplasmosis. The true seroprevalence was also lower than the overall seroprevalence in Chinese cancer patients reported from a systematic review (21%)^25^. This is however not a surprise, as cancer patients are generally older than pregnant women and thus have experienced more time at risk of infection.

The results of true seroprevalence for livestock showed that *T. gondii* infection is wide spread in meat-producing animals in different regions of China. The median of the true seroprevalences from seven regions of China in pigs (24%) was significantly higher than in cattle (9.5%), but it was not significantly higher than in chickens (20%) and small ruminants (20%). In addition, results showed that the regional variation of true seropevalences in chickens (from 11% to 24%) was strongly correlated with variation in small ruminants (Spearman correlation = 0.86, *p* = 0.01). Chickens are considered good indicators of *T. gondii* contamination in the environment, as they become infected mostly by ingesting *T. gondii* oocysts-contaminated soil^26^. Therefore, regional variability may indicate variation in environmental contamination with oocysts. In China, sheep is one of the major grazing livestock and they are grazing rotationally by following a predetermined range and routine in pastures^27^. The outdoor access of these animals means that, similarly to chickens, environmental contamination with oocysts is also major risk of infection small ruminants, which may explain the correlation between regional variations of seroprevalence in these species. However, seroprevalence is also strongly associated with farming system with the seroprevalence of *T. gondii* infection higher in outdoor farming systems than in indoor farming systems^28–30^. In addition, the age of animals, number of cats present in the farm, and feed source are considered as important risk factors associated with *T. gondii* seroprevalence^31,32^. Farming system and exposure to risk factors are likely to also vary by region, and may be correlated for the different species (e.g. in regions with a lot of backyard poultry, backyard farming may also be more common for small ruminants). Therefore, to better understand regional variability in seroprevalence it is important to collect and analyse data taking into account exposure to potential risk factors that are related to *T. gondii* infection in future studies. This type of information was lacking from most of the publications included in the review.

With the exception of cattle and horses, the risk of human *T. gondii* infection via undercooked meat likely increases with a higher seroprevalence in the animal species. Nonetheless, no strong regional relationship between the true seroprevalence in livestock and pregnant women was found. This may be due to the limited data points in our analysis (7 regions). More importantly, the total meat consumption volume and species-specific preparation habits play crucial roles for the relative attribution of different meat-producing animals on a population level^33^. In China, pork is the dominant type of meat consumed by the whole population, except for some religious groups, followed by poultry, beef and lamb, the average annual consumption volumes in 2015 were 20.1, 8.4, 1.6 and 1.2 kg respectively^34^. The highest seroprevalence and total amount of consumption make pork a good potential source of human infections. Additionally, people living in Yunnan, Guizhou and Sichuan provinces eat raw or undercooked pork and beef in their tradition, and the seroprevalence in the ethnic groups from these provinces were found higher than the general population^35^. In our study, the true seroprevalence in pregnant women from the same region was found to be one of the highest 4.8% (1.7–14%). Due to the diversification of food sources and preparation habits among different regions and ethnic groups, the risk of human infection can vary accordingly. To determine the risk of humans to become infected via the different meat-producing animals, information on prevalence in livestock and meat consumption needs to be combined in a quantitative risk assessment^36^.

In conclusion, the seroprevalence and geographical distribution of *T. gondii* infection in pregnant women and meat-producing animals in China were systematically reviewed and summarized. The results obtained from Bayesian hierarchical models showed that *T. gondii* seroprevalence ranged from 2.4% to 5.0% in pregnant women, pigs (20–33%) and chickens (11–24%) had higher true seroprevalence than small ruminants (15–24%) and cattle (6.8–12%). Studies to better evaluate the performance of kits are needed to get more accurate estimates of *T. gondii* seroprevalence in humans and animals. More detailed insight in the geographical distribution of *T. gondii* prevalence in humans and livestock animals can be helpful for making effective intervention strategies to reduce the burden of this disease in the Chinese population. However, the risk of meatborne *T. gondii* infection in humans is not only depending on the prevalence in meat-producing animals but also on consumed volumes and food preparation habits. Therefore the results obtained from this study should be used to determine meatborne toxoplasmosis risk by using a quantitative microbial risk assessment of *T. gondii* infection in China.

## Methods

### Literature review and data sources

Relevant studies on the seroprevalence of *T. gondii* infection in pregnant women and farm animals in the mainland of China were searched through the Ovid Medline, Scopus, Embase and China National Knowledge Infrastructure (CNKI) electronic databases for English and Chinese publications. Cochrane guidelines and European Food Safety Authority (EFSA) guidance for carrying out systematic reviews were followed for identifying eligible studies^37,38^, and the PRISMA guidelines was followed for reporting^39^. The key elements of this review question were: population (pregnant women and meat-producing animals in China) and outcome (*T. gondii* seroprevalence). The literature search on Medline, Scopus, Embase was last updated on 20/10/2017, and last updated on CNKI on 18/05/2016.

### Search strategy and study selection

All population-based studies published in the last 16 years (2000–2016) that reported the seroprevalence of anti-*T. gondii* antibodies in Chinese pregnant women or at least one of the animal species of interest were considered for inclusion. There was no restriction on language and the sample size of the study. The combination of “*Toxoplasma*”, “*gondii*”, “toxoplasmosis”, “China”, and “Chinese” were used as search terms in Medline, Scopus and Embase, the combination of “*T. gondii*”, “toxoplasmosis”, “pig”, “cattle”, “sheep” and “goat”, “chicken”, “duck”, “goose” and “donkey” in Chinese were searched in CNKI. One reviewer (HD), fluent in English and Chinese, read the titles and abstracts of all publications retrieved from the electronic databases and excluded those that clearly did not meet the aforementioned selection criteria. In the next stage, all potential eligible studies were retrieved in full-text and checked by the same reviewer.

Several exclusion criteria were used to select eligible studies: (a) Descriptive studies, reviews, case reports, editorials or letters to the editors without original data, individual animal diagnosis and treatments (epidemiological cross-sectional studies were preferred). (b) Studies not representative for our target population. (c) Animal species not intended for meat consumption (e.g. animals for research purpose). (d) Studies limited to experimental infection with *T. gondii* (rather than natural infection). (e) The outcomes of the studies (*T. gondii* infection) were not confirmed by a serological assay. (f) Information about the total number of seropositive samples and sample size were not available. (g) Studies not conducted in the mainland of China. (h) Duplicated data.

Information from all relevant studies was extracted and coded into a Microsoft Excel datasheet. Variables extracted included author information, year of publication, animal species, period of data collection, location of the study, diagnostic method, seroprevalence or number of seropositive cases and sample size. If a publication contained multiple studies (e.g. different livestock species or regions), data were extracted separately. For pregnant women, many studies additionally reported the prevalence of IgM antibodies, but only data on IgG seroprevalence of *T. gondii* were included in the analysis.

### Data analysis

#### Hierarchical meta-analysis model

The 31 provinces of mainland China were categorized into seven regions, including central, east, north, northeast, northwest, south and southwest of China in our study. Apparent seroprevalence in pregnant women and livestock from different regions were summarized using a three-level hierarchical meta-analysis model. First, at the individual study level, the number of seropositive samples (${x}_{i}$) out of total number of tested samples (${n}_{i}$) in every single study $i$ was assumed to follow a binomial distribution, equation (1). The logit-transformed seroprevalence ($A{P}_{i}$) of every individual study, conducted in a certain region $j$, was assumed to arise from a normal distribution with a region-specific mean seroprevalence (${\theta }_{j}$) and within-region variance (${\sigma }_{w}^{2})$, equation (2). Second, at the regional level, every specific regional seroprevalence was assumed to arise from a normal distribution with the mean of national level seroprevalence (${\theta }_{0}$) and between-region variance (${\sigma }_{b}^{2}$), equation (3).

1 $${x}_{i} \sim {\rm{Binomial}}(A{P}_{i},{n}_{i})$$

2 $${\rm{logit}}(A{P}_{i}) \sim {\rm{Normal}}({\theta }_{j},{\sigma }_{w}^{2})$$

3 $${\theta }_{j} \sim {\rm{Normal}}({\theta }_{0},{\sigma }_{b}^{2})$$

After fitting this hierarchical random effects model to the available data, seroprevalence values for regions with no data were imputed based on the resulting posterior predictive distributions. In other words, we represented missing seroprevalence data by distributions based on the fitted mean and variance parameters. For regions where no data were available, the (logit-transformed) seroprevalence was imputed as multiple random draws from a normal distribution with mean equal to the fitted national intercept ${\theta }_{0}$ and variance equal to the fitted between-region variance (thus imputing the seroprevalence as that of a “random” region, with the uncertainty interval describing the variability between regions). The equation was:

4 $${\rm{logit}}({\theta }_{j}^{\ast }) \sim {\rm{Normal}}({\hat{\theta }}_{0},{\hat{\sigma }}_{b}^{2})$$

### True seroprevalence estimation

All studies included in the analysis reported apparent seroprevalence instead of true seroprevalence. Therefore, publications about the Se and Sp for each commercial kit used in our selected studies were searched and reviewed. To take the uncertainty of Se and Sp into account, the information was then used by fitting beta distributed priors. The beta distribution is defined on the interval 0–1 and it is very flexible, therefore often used for model probabilities in Bayesian analyses. For those commercial kits which had more than one reported Se and Sp, the numbers of positive sample and tested sample obtained from validation studies were fitted to a random effect meta-analysis similar to the previous hierarchical meta-analysis model but without the multilevel structure. For those commercial kits which had no information at all, they were all categorized as unknown kit. To fit the random effect meta-analysis, data from all the known kits which were as the same type of assay as the unknown kits were used. The parameters of beta distribution were then found by using “fitdist” function in the R package “fitdistrplus” with the generated data from the random effect meta-analysis. As initial runs of the Bayesian hierarchical model showed insufficient convergence, beta distributions for the priors of Se and Sp were truncated (Table 3) assuming that the values of Se and Sp of used kits were at least larger than 0.1. The characteristics of the diagnostic tests (${Se}$ and ${Sp}$) were taken into account in equation (6), where ${{AP}}_{i}$ is the apparent seroprevalence found in the record and $T{P}_{i}$ is the true seroprevalence. The equations for the Bayesian hierarchical model were:

5 $${x}_{i} \sim {\rm{Binomial}}(A{P}_{i},{n}_{i})$$

6 $$A{P}_{i}=S{E}_{i}\ast T{P}_{i}+(1-S{P}_{i})\ast (1-T{P}_{i})$$

7 $${\rm{logit}}(T{P}_{i}) \sim {\rm{Normal}}({\pi }_{j},{\sigma }_{w}^{2})$$

8 $${\pi }_{j} \sim {\rm{Normal}}({\pi }_{0},{\sigma }_{b}^{2})$$

Statistical analyses were performed using R version 3.3.1^40^. The model was implemented in a Bayesian framework, using independent normal distributed (0, 100000) priors for all ${\theta }_{j}$ and ${\theta }_{0}$; a uniform distributed (0, 10) prior for ${\sigma }_{w}^{2}$; and a Folded-t (1) distributed prior for ${\sigma }_{b}^{2}$, as suggested by Gelman^41^. Models were constructed using “rjags” package and data were fitted using Markov Chain Monte Carlo sampling techniques. The models were implemented in R with the first 10,000 iterations as burn-in and 10,000 iterations as posterior inference. The convergence was checked by visual inspection of density and trace plots, as well as the multivariate potential scale reduction factors (or Brooks-Gelman-Rubin diagnostic). Approximate convergence was diagnosed if the upper confidence limit of the potential scale reduction factor was close to one.

The unit of analysis was set as a single study. Separate analysis was performed for pregnant women and each animal species. The estimates of posterior mean from different regions of China together with the 95% posterior probability intervals, defined as the distribution’s 2.5^th^ and 97.5^th^ percentile, were generated from the analysis. A Kruskal-Wallis test was used to compare the true seroprevalences in different species. If the Kruskal-Wallis test showed a significant result then a post-hoc analysis will be performed using Dunn’s test and *p*-values were adjusted by “Holm” method for multiple comparisons. The relationship between seroprevalence in pregnant women and meat-producing animals in different regions of China was estimated by checking Spearman’s rank correlation coefficient.

### Data availability

The datasets generated during and/or analysed during the current study are available from the corresponding author on reasonable request.

## Electronic supplementary material

Supplementary file S1

Supplementary S2
