# Supplementary material for: Seroprevalence of Toxoplasma gondii in pregnant women and livestock in the mainland of China: a systematic review and hierarchical meta-analysis
Source: Sci Rep. 2018 Apr 18;8:6218. doi: 10.1038/s41598-018-24361-8 (PMC5906581; doi:10.1038/s41598-018-24361-8)
Supplement: Supplementary file 1 — Supplementary file S1 [file 41598_2018_24361_MOESM1_ESM.pdf]

Seroprevalence of *Toxoplasma gondii* in pregnant women and livestock in the mainland of China: a systematic review and hierarchical meta-analysis

Huifang Deng[1], Brecht Devleesschauwer[2], Mingyuan Liu[3], Jianhua Li[3], Yongning Wu[4], Joke van der Giessen[1], and Marieke Opsteegh[1,*]

[1] Centre for Infectious Disease Control - Zoonoses and Environmental Microbiology, National Institute for Public Health and the Environment, 3720 BA, Bilthoven, The Netherlands

[2] Department of Public Health and Surveillance, Scientific Institute of Public Health (WIV-ISP), 1050, Brussels, Belgium

[3] Institute of Zoonosis, Jilin University, 130062, Changchun, People's Republic of China

[4] National Center for Food Safety Risk Assessment, Key Laboratory of China Food Safety Risk Assessment, 100022, Beijing, People's Republic of China

* Corresponding author
E-mail: marieke.opsteegh@rivm.nl (MO)

Table S1. Summary of total selected papers for pregnant women and livestock.

Population	No. of selected papers
Pregnant women	72
Chicken	20
Cattle	38
Pig	80
Small ruminant	51
Duck	5
Goose	5
Donkey	5

Selected papers for pregnant women in mainland of China

[1]王英,杨文丽.河北省霸州市孕妇弓形虫感染及其知晓度的相关性调查[J].中国血吸虫病防治杂志,2016:1-3.
[1] Wang, Y., Yang, W. Prevalence and awareness of *Toxoplasma gondii* of pregnant women in Bazhou City, Hebei Province. *Chin. J. Schisto. Control.* 1-3 (2016).

[2]庞艳,韩卫全.妊娠 20 周前孕妇 TORCH 感染情况及相关因素的分析[J].中国妇幼保健,2016,08:1596-1598.
[2] Pang, Y., Han, W. Prevalence of TORCH and risk factor analysis. *Maternal and Child Health Care of China*. **8**, 1596-1598 (2016).

[3]周佼佼,陶莉莉.无锡地区孕妇弓形虫感染及危险因素调查[J].中国血吸虫病防治杂志,2015,06:604-607.
[3] Zhou, J., Tao, L. Seroprevalence and risk factors of *Toxoplasma gondii* infection among pregnant women in Wuxi region. *Chin. J. Schisto. Control.* **27**, 604-607 (2015).

[4]陶芳,陈博,秦文燕.巢湖地区 5028 例孕早期妇女 TORCH 感染回顾性分析[J].中国优生与遗传杂志,2016,01:85-86.
[4] Tao, F., Chen, B., Qin, W. Retrospective analysis of TORCH infection of 5028 women in the early period of pregnancy in area of Chaohu. *Chinese J. of Birth Health and Heredity*. **1**, 85-86 (2016)

[5]丁睿,张智贤,曾华,陈梅,王伦善.6027 例孕产妇及新生儿 TORCH 检测结果分析[J].国际检验医学杂志,2015,04:485-486.
[5] Ding, R., Zhang, Z., Zeng, H., Chen, M. Wang, L. Analysis on TORCH test in 6027 cases of pregnant women, ouerperae and neonates. *Int. J. Lab. Med.* **4**, 485-486 (2015).

[6]石伟娟,徐秀叶,潘继美,雷云娟,杜晓东.孕妇 TORCH 感染筛查结果分析[J].中华医院感染学杂志,2015,04:925-927.
[6] Shi, W., Xu, X., Pan, J., Lei, Y., Du, X. Analysis of TORCH infection screening in pregnant women. *Chin. J. Nosocomiol*. **4**, 925-924 (2015).

[7]侯丽萍,张慧芳,饶华祥,赫燕侠,侯红娟,崔爱永,侯玉英.太原市孕妇弓形虫感染现况调查[J].中国病原生物学杂志,2015,01:100-101.
[7] Hou, L. et al. A cross-sectional survey of *Toxoplasma gondii* infection in pregnant women of Taiyuan. *J. of Pathogen Biology*. **1**, 100-101 (2015).

[8]林颖悦,许妹珍,郭俊杰.齐齐哈尔市孕妇弓形虫感染情况调查[J].齐齐哈尔医学院学报,2015,15:2275-2276.
[8] Lin, Y., Xu, M., Guo, J., Survey on *Toxoplasma gondii* infection in pregnant women in Qiqihar. *J. of Qiqihar University of Medicine.* **15**, 2275-2276 (2015)

[9]康栗.安徽淮南地区孕妇弓形虫感染情况调查[J].热带病与寄生虫学,2015,02:108-109.
[9] Kang, L. Survey on *Toxoplasma gondii* infection in pregnant women in Huainan, *Anhui. J. of Tropical Disease and Parasitology*. **2**, 108-109 (2015).

[10]谢小娟,李小侠,李芒会,张利侠.西安地区 9338 例孕妇 TORCH 检测结果分析[J].医学综述,2015,16:3016-3018.
[10] Xie, X., Li, X., Li, M., Zhang, L. Analysis of 9338 cases of TORCH detection in pregnant women in Xi'an area. *Medical Recapitulate*. **16**, 3016-3018 (2015).

[11]吴佳丽,朱建昌.365 例妊娠期妇女 TORCH 感染的血清学检测及妊娠结局分析[J].中国微生态学杂志,2015,10:1218-1220.
[11] Wu, J., Zhu, J. Serological detection of TORCH in 365 pregnant women and pregnancy outcome analysis. *Chinese J. of Microecology*. **10**, 1218-1220 (2015).

[12]王琳,陈秀兰,袁学华,罗卉丽.31275 例孕妇 TORCH 检测结果分析[J].湖北医药学院学报,2015,04:399-400.
[12] Wang, L., Chen, X., Yuan, X., Luo, H. Analysis of TORCH detection outcome in 31275 pregnant women. *J. of Hubei University of Medicine*. **4**, 399-400 (2015).

[13]黄蕾,姚建强,李文瑛,史春妮,杨品胜,朱艳,王芳.陕西地区孕妇 TORCH 感染指标检测结果分析及临床意义探讨[J].国际检验医学杂志,2015,21:3191-3193.
[13] Huang, L. et al. Analysis of TORCH detection outcome in pregnant women in Shanxi area and discussion of clinical significance. *Int. J. Lab. Med.* **21**, 3191-3193 (2015).

[14]辛克盛,潘敏,刘慧,董荣荣.青岛地区孕妇弓形虫感染调查[J].中国血吸虫病防治杂志,2014,03:320-322.
[14] Xin, K., Pan, M., Liu, H., Dong, R. Investigation of *Toxoplasma gondii* infection in Qingdao area. *Chin. J. Schisto. Control.* **3**, 320-322 (2014).

[15]陈玉昆,杨亚晓,魏世锦,宋任浩.孕早期妇女弓形虫感染对妊娠结局的影响[J].中国血吸虫病防治杂志,2014,03:308-310.
[15] Chen, Y., Yang, Y., Wei, S., Song, R., Impact of *Toxoplasma gondii* infection on pregnant outcomes in early pregnant women. *Chin. J. Schisto. Control.***3**, 308-322 (2014).

[16]赵聪伟.安国市孕妇弓形虫感染状况及其对妊娠结局的影响[J].热带医学杂志,2014,08:1057-1059.
[16] Zhao, C. Impact of Toxoplasma gondii infection on pregnancy outcomes in pregnant women. J. Trop. Med. 8, 1057-1059 (2014).

[17]张慧芳,侯玉英,杨晓丽,王泽波.太原市部分孕妇感染弓形虫状况的调查与分析[J].中国药物与临床,2014,10:1367-1368.
[17] Zhang, H., Hou, Y., Yang, X., Wang, Z. Survey of *Toxoplasma gondii* infection in pregnant women in Taiyuan. *Chinese Remedies & Clinics*. **10**, 1057-1059 (2014).

[18]贺娜珍,刘丽萍,胡碧燕.普陀区围孕期妇女 TORCH 感染筛查结果分析[J].中华医院感染学杂志,2013,21:5231-5232.
[18] He, N., Liu, L., Hu, B. Screening of TORCH infection in periconceptional women in Putuo area. *Chin. J. Nosocomiol*. **21**, 5232-5235 (2013).

[19]丁舫,王榴红.孕妇血清 TORCH 四种病原体感染血清学筛选的临床分析[J].放射免疫学杂志,2013,06:821-822.
[19] Ding, F., Wang, L. Screening of TORCH infection in pregnant women and clinical analysis. *J. of Radioimmunology*. **6**, 821-822 (2013).

[20]夏玲,黄月新.无锡地区孕妇弓形虫感染情况调查[J].热带病与寄生虫学,2013,03:169-170.
[20] Xia, L., Huang, Y. Survey of *Toxoplasma gondii* infection in pregnant women in Wuxi area. *J. of Tropical Disease and Parasitology*. **3**, 169-170 (2013).

[21]张欠欠,成俊珍,王逢会.延安市 2850 例孕妇弓形虫感染情况调查[J].中国病原生物学杂志,2013,03:292-293.
[21] Zhang, Q., Cheng, J., Wang, F. Study on toxoplasmosis among 2850 pregnant women in Yan'an. *J. of Pathogen Biology*. **3**, 292-293 (2013).

[22]龙聪,范文,张家钧,刘学政.荆州地区 3851 例孕妇 TORCH 感染的调查分析[J].中国妇幼保健,2013,16:2579-2581.
[22] Long, C., Fan, W., Zhang, J., Liu, X. Investigation on TORCH infection of 3851 cases of pregnant women in Jingzhou district. *Maternal and Child Health Care of China*. **16**, 2579-2581 (2013).

[23]方斐,王红贤.优生优育 TORCH 筛查 889 名孕妇结果分析[J].实用医技杂志,2013,06:609-610.
[23] Fang, F., Wang, H. Analysis of TORCH infection outcome in 889 pregnant women. *J. of Practical Medical Techniques*. **6**, 609-610 (2013).

[24]秦永超,岳红霞,王彬,黄丽华,尹继刚,向梅.长春地区孕妇感染弓形虫株的基因分型[J].中国病原生物学杂志,2013,09:790-792.
[24] Qin, Y. et al. Genotyping of *Toxoplasma gondii* isolates from pregnant women in Changchun. *J. of Pathogen Biology*. **9**, 790-792 (2013).

[25]池新安,肖冬珍.生育期妇女与弓形虫感染关系的探讨[J].临床医药实践,2012,01:34-35.
[25] Chi, X., Xiao, D. The investigation of the relationship between bearing women and *Toxoplasma gondii* infection. *Proceeding of Clinical Medicine*. **1**, 34-35 (2012).

[26]陈文举,朱杰,徐友文,吴晓宇,李招云.台州地区 5613 例孕早期妇女 TORCH 三项病原体感染情况调查分析[J].中国卫生检验杂志,2012,04:797-798.
[26] Chen, W., Zhu, J., Xu, Y., Wu, X., Li, Z. The investigation and analysis of TORCH infection of 5613 women in the early period of pregancy in Taizhou. *Chinese J. of Health Laboratory Technology*. **4**, 797-798 (2012).

[27]杜宝琴,王新英,刘淑敏,薛连才.超声筛查孕妇孕早期弓形虫感染致出生缺陷分析[J].中国优生与遗传杂志,2012,06:88+125.
[27] Du, B., Wang, X., Liu, S., Xue, L. Analysis of birth defects induced by Toxoplasma gondii infection in pregnant women in early pregnancy by ultrasound screening. *Chinese J. of Birth Health and Heredity*. 6, 88-89(2012).

[28]何桂儿,曾华,王晶晶,黄霜,王蕴端,黄志成.孕妇血清中抗弓形虫抗体 IgG 和 IgM 检测分析[J].临床医学工程,2012,06:956-957.
[28] He, J. et al. The detection analysis of anti-Toxoplasma antibodies IgG and IgM in maternal serum. Clinical Medicine and Engineering. 6, 956-957 (2012).

[29]索庆丽,刘胜武,姚婷.武汉市弓形虫 IgM 阳性孕妇异常妊娠结局研究[J].现代预防医学,2011,05:863-865.
[29] Suo, Q., Liu, S., Yao, T. Study of abnormal pregnancy outcome among toxoplasma IgM-positive pregnant women in Wuhan. Modern Preventive Medicine. 5, 863-865 (2011).

[30]丘继哲,邹艳,徐妮为,邓雪冰,黄旭.株洲市孕产妇弓形虫感染的血清学分析[J].临床合理用药杂志,2011,04:100-101.
[30] Qiu, J., Zou, Y., Xu, N., Deng, X., Huang, X. Seroprevalence of Toxoplasma gondii infection in pregnant women in Zhuzhou. Chin. J. of Clinical Rational Drug Use. 4, 100-101 (2011).

[31]夏薇,张小俊,陈锡慰.南京地区孕妇弓形虫感染不同妊娠结局研究[J].中国血吸虫病防治杂志,2011,02:183-186.
[31] Xia, W., Zhang, J., Chen, X. Investigation of different pregnant results of pregnant women infected with Toxoplasma gondii in Nanjing region. *Chin. J. Schisto. Control.* 2, 183-186 (2011)

[32]俞菁,章莉,张昕明,李俊,谭美玉,李海川,龚波.某市妇女 TORCH 感染调查分析[J].国际检验医学杂志,2011,09:984-985.
[32] Yu, J. et al. Survey on TORCH infection in pregnant women in one city. *Int. J. Lab. Med.*9, 984-985 (2011).

[33]李晓平.酶联免疫吸附试验检测孕妇弓形虫感染的临床研究[J].实用医技杂志,2011,08:808-810.
[33] Li, X. Enzyme-linked immunosorbent assay detection of Toxoplasma gondii infection of pregnant women. J. of Practical Medical Techniques. 8, 808-810 (2011).

[34]李慧萍.优生四项检测 500 例临床分析与意义探讨[J].基层医学论坛,2011,25:790-791.
[34] Li, H. Analysis and discussion for 500 cases of eugenics four examination. The Medical Forum. 25, 790-791 (2011).

[35]江婷,刘成程,陈冬梅.TORCH 感染的血清学筛查[J].沈阳医学院学报,2011,03:186-188.
[35] Jiang, T., Liu, C., Chen, D. Serological screening for TORCH infections. J. of Shenyang Medical College. 3, 186-188 (2011).

[36]黄河,梁莉琴,黄建超,张良先,朱堂意.仁寿地区孕妇弓形虫感染的血清学调查[J].中国卫生检验杂志,2010,05:1226-1227.
[36] Huang, H., Liang, L., Huang, J., Zhang, G., Zhu, T. Serological investigation of Toxoplasma infection among Renshou pregnant women. Chinese J. of Laboratory Technology. 5, 1226-1227 (2010).

[37]张立娟.孕早期弓形虫抗体检测的临床意义初探[J].检验医学与临床,2010,17:1848-1850.
[37] Zhang, L. Clinical significance of anti-Toxoplasma antibody in early pregnancy. 17, 1848-1850 (2010).

[38]唐凤,华海涌,胡一河,卫文学,吴民义.江苏省南部 3 市孕妇弓形虫感染情况调查[J].中国血吸虫病防治杂志,2010,05:436.
[38] Tang, F., Hua, H., Hu, Y., Wei, W., Wu, M., Investigation of Toxoplasma gondii infection among pregnant women in three cities of southern Jiangsu province. *Chin. J. Schisto. Control.* 5, 436 (2010).

[39]索庆丽,姚婷.武汉市孕妇弓形虫感染状况研究[J].中国妇幼保健,2009,05:598-600.
[39] Suo, L., Yao, T. Survey on Toxoplasma gondii infection in pregnant women in Wuhan. *Maternal and Child Health Care of China.* 5, 598-600 (2009).

[40]刘雁茹,郭树江.孕妇弓形虫感染的调查[J].基层医学论坛,2009,07:271-272.
[40] Liu, Y., Guo, S. Survey on Toxoplasma gondii infection in pregnant women. The Medical Forum. 7, 271-272 (2009).

[41]谢跃文,王强.250 例 TORCH 检测结果分析[J].实验与检验医学,2009,06:713.
[41] Xie, Y., Wang, Q. Analysis TORCH detection results from 250 cases. Experimental and Laboratory Medicine. 6, 271-272 (2009).

[42]郑炳友,赵安成.沂蒙山区孕妇弓形虫感染调查报告[J].社区医学杂志,2008,17:23-24.
[42] Zeng, B., Zhao, A., Survey on Toxoplasma gondii infection in pregnant women in Yimengshan area. J. of Community Medicine. 17, 23-24 (2008).

[43]周庆新,吴金华.舟山群岛孕妇弓形虫感染率及其与不良妊娠结局关系的调查[J].疾病监测,2007,02:104-106.
[43] Zhou, Q., Wu, J. The relationship between Toxoplasma gondii infection and abnormal pregnancy outcome in Zhoushan pregnant women. Disease Surveillance. 2, 104-106 (2007).

[44]余品红,陈建设,张华勋,张迟,王重新,毛重喜,徐明星.武汉地区人群弓形虫感染血清学调查分析[J].中国人兽共患病学报,2007,04:393-394.
[44] Yu, P. et al. The serological survey of Toxoplasma infection in Wuhan city. Chinese J. of Zoonoses. 4, 393-394 (2007).

[45]唐莉娜,徐莉娜,卢丽丹,李安梅,周光荣,林广初.贵州省孕妇弓形虫血清学监测及评价[J].贵州医药,2007,09:847-848.
[45] Tang, L. et al. Serological surveillance and evaluation of Toxoplasma gondii infection in pregnant women in Guizhou. Guizhou Medical Journal. 9, 847-848 (2007).

[46]张静,覃建庆,朱志洁,陈丽春,王亚婷.大庆市妊娠妇女弓形虫感染的调查分析[J].黑龙江医药科学,2006,04:69-70.
[46] Zhang, J., Qin, J., Zhu, Z., Chen, L., Wang, Y. Survey on Toxoplasma gondii infection in pregnant women in Daqin. Heilongjiang Medicine and Phamacy. 4, 69-70 (2006).

[47]张英,李卉.兰州地区孕妇弓形虫感染情况调查[J].卫生职业教育,2005,23:81-82.
[47] Zhang, Y., Li, H., Survey on Toxoplasma gondii infection in pregnant women in Lanzhou area. Health vocational Education. 23, 81-82 (2005).

[48]潘晓平.孕妇弓形虫感染的抗体检测[J].浙江实用医学,2005,02:132.
[48] Pan, X. Detection of Toxoplasma gondii antibodies in pregnant women. Zhejiang Practical Medicine. 2, 132 (2005).

[49]景立新,杜彩素,韩英,张萍,李雅杰.大连地区 93 例孕妇弓形虫感染情况调查[J].实用预防医学,2005,03:653-655.
[49] Jing, L., Du, C., Han, Y., Zhang, P., Li, Y. Survey of Toxoplasma gondii infection in 93 pregnant women in Dalian. Practical Prevention Medicine. 3, 653-655 (2005).

[50]叶华珍,古似丹,黄春琴.妊娠期 TORCH 筛查与妊娠结局探讨[J].中国初级卫生保健,2005,06:12-13.
[50] Ye, H., Gu, S., Huang, C. TORCH screenings in gestational period and outcomes of fetus from the pregnant women. Chinese Primary Health Care. 6, 12-13 (2005).

[51]冯月菊,刘小宁,任文锋,潘志明,高雨藩,郭荣同.广州市 2004 年弓形虫病血清学调查分析[J].热带医学杂志,2005,06:108-109+123.
[51] Feng, Y. et al. Serological analysis of Toxoplasmosis in Guangzhou city in 2004. J. of Tropical Medicine. 6, 108-109 (2005).

[52]骆美瑛,朱寿华,骆建萍,余慧玲.孕妇弓形虫感染血清学筛查的探讨[J].江苏预防医学,2005,04:62-63.
[52] Luo, M., Zhu, S., Luo, J., Yu, H. Serological screening and discussion on Toxoplasma gondii infection in pregnant women. Jiangsu Prev. Med. 4, 62-63 (2005).

[53]吴大富,杨红梅.1082 例早期妊娠妇女弓形虫感染筛查分析[J].实用医技杂志,2005,22:3335-3336.
[53] Wu, D., Yang, H. Screening analysis of Toxoplasma gondii infection in 1082 pregnant women. 22, 3335-3336 (2005).

[54]蒋松云,卞红珍,杨芸,周杭娟,周建华,卢丹.上海市虹口区孕妇弓形虫感染情况调查[J].上海预防医学杂志,2005,05:210-212.
[54] Jiang, S. et al. Study on Toxoplasma infection among pregnant women in Hongkou district Shanghai. Sh. J. Prev. Med. 5, 210-212 (2005).

[55]张艳霞.济宁 154 名孕妇及其新生儿弓形虫感染的调查[J].华西医学,2004,02:287-288.
[55] Zhang, Y., Survey of Toxoplasma gondii infection in 154 pregnant women and newborns. West China Medical J. 2, 287-288 (2004).

[56]陆小婵,卢冬.孕产妇 4 种病原体感染血清学筛查的临床意义[J].右江民族医学院学报,2004,04:557-558.
[56] Lu, X., Lu, D. Clinical significance of serological screening for four pathogens in pregnant women. J. of Youjiang Medical College for Nationalities. 4, 557-558 (2004).

[57]吴菊.2503 例孕妇弓形虫感染检测结果分析[J].浙江检验医学,2004,02:17+19.
[57] Wu, J. Analysis of Toxoplasma gondii infection in 2503 pregnant women. Zhejiang J. of Laboratory Medicine. 2, 17-19 (2004).

[58]贺能,吴金华.舟山群岛 1999～2002 年孕妇弓形虫感染情况调查[J].中华流行病学杂志,2003,10:71.
[58] He, N., Wu, J. Survey of Toxoplasma gondii infection in pregnant women in Zhoushan during 1999-2002. Chin. J. Epidemiol. 10, 71 (2003).

[59]李昭荣,李牧,刘海莉,杜芳,刘明,赵娟.分娩期孕妇 TORCH 感染的血清学检测及临床分析[J].国外医学.妇幼保健分册,2003,01:3-5.

[59] Li, Z., et al, The examination and analysis of serum TORCH infection during labor stage. Foreign Medical Sciences. 1, 3-5 (2003).

[60]刘琦,姚莉萍.绍兴市 15655 例育龄妇女和孕妇中弓形虫感染血清学分析[J].江西医学检验,2003,06:501-484.
[60] Liu, Q., Yao, L. Serological analysis of Toxoplasma gondii infection in 15655 women of childbearing age and pregnant women in Shaoxin. Jiangxi J. of Medical Laboratory Sciences. 6, 501 (2003).

[61]陈华明,乐周萍,柴辉,吴文权.黄石地区 2845 例孕妇弓形虫感染的血清学检测[J].实用寄生虫病杂志,2002,02:59.
[61] Chen, H., Le, Z., Cai, H., Wu, W. Serological detection of Toxoplasma gondii infection in 2845 pregnant women in Huangshi area. J. of Practical Parasitic Disease. 2, 59 (2002).

[62]刘学宁,林敏,高世同,占国清.深圳市有关人群弓形虫感染的血清学流行病学调查研究[J].中国公共卫生管理,2001,04:320-321.
[62] Liu, X., Lin, M., Gao, S., Zhan, G. Seroepidemiology study on Toxoplasma gondii infection in Shenzhen city. Chin. J. of PHM. 4, 320-321 (2001).

[63]林睦成,翁秋桂,肖秀容,蔡戈燕,宁新民,吕玲.预婚女青年与孕妇弓形虫感染特征初步研究[J].中国优生与遗传杂志,2001,04:100-110.
[63] Lin, M., et al. Preliminary study on Toxoplasma gondii infection in premarital women and pregnant women. *Chinese J. of Birth Health and Heredity.* 4, 100-110 (2001).

[64]陈新祥,章顺婷.绍兴市孕妇弓形虫感染的血清学调查[J].疾病监测,2000,04:140-141.
[64] Chen, X., Zang, S. Serological survey on Toxoplasma gondii infection in pregnant women in Shaoxin. Disease Surveillance. 4, 140-141 (2000).

[65]刘惠莲.南京市大厂区部分人群弓形虫感染血清学调查[J].预防医学文献信息,2000,01:18-19.
[65] Liu, H. Serological survey of Toxoplasma gondii infection in Dachang district, Nanjing. Lit. Inf. Prev. Med. 1, 18-19 (2000).

[66]李懿宏, 努瓦克肖特市区孕妇弓形虫感染状况调查[J]. 中国动物学会第八次全国寄生虫学学术讨论会论文摘要汇编,2001,2.
[66] Li, Y. Survey on Toxoplasma gondii infection in pregnant women in Nuwakexiaote. 8th National Parasitology Symposium. 2, (2001).

[67]高红艳. 孕妇感染性疾病筛查 5360 例临床分析[J]. 基层医学论坛,2013,05:601-603.
[67] Gao, Y. Sceening of infectious disease in 5360 pregnant women. The Medical Forum. 5, 601-605 (2013).

[68]梁多宏,王凤芝,范红,韩松,段玲,崔策. 孕妇弓形虫感染的血清流行病学分析[J]. 中国初级卫生保健,2004,02:30-31.
[68] Liang, D. et al. Seroepidemiology of Toxoplasma gondii infection. Chinese Primary Health Care. 2, 30-31 (2004).

[69]马玉燕,牟瑞丽,王磊一,江森. 孕妇与胎儿弓形虫感染状况的研究[J]. 中国寄生虫病防治杂志,2002,05:24-26.

[69] Ma, Y., Mou, R., Wang, L., Jiang, S. Study on Toxoplasma gondii infection in pregnant women and fetus. Chin. J Parasit. Dis. Con. 5, 24-26 (2002).

[70]韩宁宁,夏燕,刘志娟,段玲.孕妇产前八项检测 1706 例结果分析[J].中国优生与遗传杂志,2014,08:68+70.
[70] Han, N., Xia, Y., Liu, Z., Duan, L. Analysis of results from eight tests in 1706 pregnant women. *Chinese J. of Birth Health and Heredity*. 8, 68-70 (2014).

[71] Cong, W. *et al.* Toxoplasma gondii Infection in Pregnant Women: A Seroprevalence and Case-Control Study in Eastern China. *Biomed Res Int* **2015**, 170278, doi:10.1155/2015/170278 (2015).

[72] Liu, H., Xin, K. S., Jiang, Y. H. & Jiang, Z. Y. Seroprevalence of Toxoplasma gondii infection among pregnant women in Shandong Province, China. *Research Journal of Medical Sciences* **8**, 42-44, doi:10.3923/rjmsci.2014.42.44 (2014).

Selected papers for pig in mainland of China

[1]何彦春,李珊,李旭蓉,钱振波.甘肃省张掖市动物弓形虫病血清学调查[J].中国动物检疫,2016,03:12-13.
[1] He, Y., Li, S., Li, X., Qian, Z. Serological survey of animal Toxoplasmosis in Zhangye city or Gansu province. China Animal Health Inspection. 3, 12-13 (2016).

[2]杨晓伟,赵光伟.重庆地区不同饲养阶段猪弓形虫感染情况的调查与分析[J].中国兽医杂志,2015,02:44-45.
[2] Yang, X., Zhao, G. Study on Toxoplasma gondii infection in pigs from different feeding stages in Congqin. Chinese J. of Veterinary Medicine. 2, 44-45 (2015).

[3]游锦洲,张瑞振,丘建龙,谢敏,胡玲英.福建省猪弓形虫病流行病学调查报告[J].当代畜牧,2015,06:22-23.
[3] You, J., Zhang, R., Qiu, J., Xie, M., Hu, L. Seroeprevalence of Toxoplasma gondii infection in swine in Fujian province. Morden Animal Husbandry. 6, 22-23(2015).

[4]温青娜,郭延华,杨菊凤,申红,杜迎春.河南省猪弓形虫感染血清学调查[J].中国兽医杂志,2015,04:44-45.
[4] Wen, Q., Guo, T., Yang, J., Shen, H., Du, Y. Seroprevalence of Toxoplasma gondii infection in pigs in Henan province. Chinese J. of Veterinary Medicine. 4, 44-45 (2015).

[5]尹杨莎,黎桂云,吴永银,董保豫,王家明,蒙炳超.黔南州猪弓形虫病血清学调查[J].当代畜牧,2015,11:81-82.
[5] Yin, Y., Li, G., Wu, Y., Dong, B., Wang, J., Meng, B. Seroprevalence of Toxoplasma gondii infection in pigs in Qiannan. 11, 81-82 (2015).

[6]金安才仁.青海省称多县猪弓形虫感染血清学流行病调查[J].黑龙江畜牧兽医,2015,16:110-111.
[6] Jin, A. Seroprevalence of Toxoplasma gondii infection in pigs in Chengduo, Qinghai. Heilongjiang Animal Science and Veterinary Medicine. 16, 110-111 (2015).

[7]刘俐君,田益明,张见,赵小波,林洁.达州市猪弓形虫病血清学调查研究[J].中国动物保健,2015,09:72-74.
[7] Liu, L., Tian, Y., Zhang, J., Zhao, X., Lin, J. Serological investigation of Toxoplasma gondii infection in pigs in Dazhou. Zhongguo Dongwu Baojian, 9, 72-74 (2015).

[8]刘辉兰.武平县猪、牛、羊弓形虫病的血清学调查[J].福建畜牧兽医,2014,02:16-18.
[8] Liu, H. Serological investigation of Toxoplasma gondii infection in pigs, cattle and small ruminants in Wupin. Fujian J. of Animal Husbandry and Veterinary Medicine. 2, 16-18 (2014).

[9]肖芳萍,余波,史开志,杨粤凯,杨茂生,徐洪忠,任荣清.贵阳地区猪弓形虫病的血清学调查与分析[J].畜牧与兽医,2014,08:86-88.
[9] Xiao, F. et al. Serological investigation of Toxoplasma gondii infection in pigs in Guiyang area. Animal Husbandry & Veterinary Medicine. 8, 86-88 (2014).

[10]陆桂丽,王文,陈卫东,汪萍,沙依兰·卡依扎,成进,夏俊.新疆部分地区猪弓形虫抗体监测[J].动物医学进展,2014,07:125-127.
[10] Lu, J. et al. Surveillance of Toxoplasma gondii infection in pigs in Xinjiang areas. Progress in Veterinary Medicine. 7, 125-127 (2014).

[11]邵晓冬,袁林,翟凯,钱伟锋,闫文朝,王天奇.洛阳地区猪弓形虫病血清流行病学调查和 PCR 检测[J].黑龙江畜牧兽医,2014,16:75-77.
[11] Serological investigation and PCR detection of Toxoplasma gondii infection in pigs in Luoyang area. Heilongjiang Animal Science and Veterinary Medicine. 16, 75-77 (2014).

[12]顾冬花,李涵林.猪弓形虫病的血清学检测[J].山东畜牧兽医,2014,07:53-54.
[12] Gu, D., Li, H. Serological detection of Toxoplasma gondii infection in pigs. Shandong J. of Animal Science and Veterinary Medicine. 7, 53-54 (2014).

[13]陈淑芳.宁波某地猪弓形虫病的流行病学及防控措施调查[J].浙江畜牧兽医,2014,05:63-66.
[13] Chen, S. Epidemiological survey on Toxoplasma gondii infection in pigs in Ningbo area. Zhejiang J. of Animal Science and Veterinary Medicine. 5, 63-66 (2014).

[14]徐斌,张夏兰,董春霞,冯超,苏兵,何琴,曾政.重庆地区猪弓形虫病流行病学调查研究[J].动物医学进展,2013,03:37-40.
[14] Xu, B. et al. Epidemiological survey on Toxoplasma gondii infection in pigs in Congqin area. Progress in Veterinary Medicine. 3, 37-40 (2013).

[15]翟少钦,付文贵,郑华,付利芝.川渝两地猪弓形体血清流行病学调查[J].中国兽医杂志,2013,02:49-50.
[15] Qu, S., Fu, W., Zeng, H., Fu, L. Epidemiological survey on Toxoplasma gondii infection in pigs in Chuan and Yu area. Chinses J. of Veterinary Medicine. 2, 49-50 (2013).

[16]贡嘎,普琼,落桑阿旺,米玛,索朗斯珠.西藏部分地区藏猪弓形虫血清学调查研究[J].中国畜牧兽医文摘,2013,02:126+158.
[16] Gong, G., Pu, Q., Luo, S., Mi, M., Suo, L. Epidemiological survey on Toxoplasma gondii infection in pigs in Tibet. Chinese Abstracts of Animal Husbandry and Veterinary Medicine. 2, 126 (2013).

[17]蒋法成,蒋锁俊,陈树霞,孙浩.淮安市猪弓形虫病血清学调查[J].上海畜牧兽医通讯,2013,02:47+49.

[17] Epidemiological survey on Toxoplasma gondii infection in pigs in Huaian. Shanghai J. of Animal Husbandry and Veterinary Medicine. 2, 47 (2013).

[18]刘琨.信阳地区猪弓形虫病血清学调查与分析[J].河南农业科学,2013,05:166-168.
[18] Liu, Q. Serological survey of the infection of swine toxoplasmosis in Xinyang. J. of Henan Agricultural Sciences. 5, 166-168 (2013).

[19]毛以智,王兴群,赵福葳,伍波涛,陈能桥,徐全忠.遵义市部分规模养猪场 7 种疫病的血清学调查[J].安徽农业科学,2013,12:5366+5369.
[19] Mao, Y. et al. Serological survey on seven diseases in pig farms in Zuenyi. J. of Anhui Agri. Sci. 12, 5366-5369 (2013).

[20]雷程红,郭芙莉,王伊丽,包振中,蔡元庆.乌鲁木齐地区猪犬鸡弓形虫病血清学调查[J].新疆畜牧业,2013,11:28-30.
[20] Lei, C., Guo, F., Wang, Y., Bao, Z., Cai, Y. Serological survey on Toxoplasma gondii infection in pigs and chicken in Wulumuqi. XinJiang Xu Mu Ye. 11, 28-30 (2013).

[21]王海霞.湟中县猪弓形虫病血清学调查[J].中国动物检疫,2012,02:44-45.
[21] Wang, H. Serological survey on Toxoplasma gondii infection in pigs in Huangzhong. China Animal Health Inspection. 2, 44-45 (2012).

[22]孔猛,白昀,丁志勇,刘茂军,冯志新,熊祺琰,白方方,车巧林,王海燕,王占伟,韦艳娜,邵国青.江苏省部分地区动物弓形虫病血清学调查[J].江苏农业科学,2012,02:171-173.
[22] Kong, M. et al. Serological survey on Toxoplasma gondii infection in animals in Jiangsu. Jiangsu Agriculture Science. 2, 171-172 (2012).

[23]蔡进忠,李春花.青海家畜弓形虫病血清学调查与流行情况分析[J].中国兽医杂志,2011,01:45-46.
[23] Cai, J., Li, C. Epidemiological survey on Toxoplasma gondii infection in livestock in Qinghai. 1, 45-46 (2012).

[24]王为升,张金生,陈伟,王俊伟,孟庆玲,乔军.石河子地区人畜弓形虫感染的血清学调查[J].动物医学进展,2011,02:120-122.
[24] Wang, W. et al. Seroprevalence of Toxoplasma gondii infection in human and animals in Shihezi area. 2, 120-122 (2011).

[25]韩建强,孙秀涛,李兵,段纲,邹丰才.云南省玉溪市猪弓形虫病血清学调查[J].黑龙江畜牧兽医,2011,07:102-103.
[25] Han, J., Sun, X., Li, B., Duan, G., Zou, F. Seroprevalence of Toxoplasma gondii infection in pigs in Yuxi, Yunnan province. Heilongjiang Animal Science and Veterinary Medicine. 7, 102-103 (2011).

[26]蒋云,邬良贤.龙岩市新罗区部分猪场弓形虫感染的血清学调查[J].福建畜牧兽医,2011,02:6-7.
[26] Jiang, Y., Wu, L. Seroprevalence of Toxoplasma gondii infection in pigs in Xinluo, Longyan. Fujian J. of Animal Husbandry and Veterinary Medicine. 2, 6-7 (2011).

[27]王军,单俊娟,蒋文生,骆志强,郑朝峰,顾曦,周华萍.农三师猪弓形虫病血清学调查与分析[J].中国动物检疫,2011,04:62-63.
[27] Wang, J. et al. Seroprevalence of Toxoplasma gondii infection in pigs in Nongsanshi. China Animal Health Inspection. 4, 62-63 (2011).

[28]李建萍.西山区 5 种动物血清弓形虫抗体调查研究[J].中外医疗,2011,24:125.
[28] Li, J. Survey on Toxoplasma gondii infection in 5 animals in Xishan district. 24, 125 (2011).

[29]向忠菊,段永兰,岳新军,陈鹏举.安徽部分地区猪弓形虫病的血清学调查研究[J].湖南农业科学,2011,17:134-135+138.
[29] Xiang, Z., Duan, Y., Yue, X., Chen, P. Serological investigation of Swine Toxoplasmosis in partial area of Anhui province. Hunan Agriculture Sciences. 24, 125 (2011).

[30]刘敏,万宝云,魏建宏,舒凡帆,杨建发,邹丰才.云南藏猪弓形虫病血清学调查[J].中国畜禽种业,2011,12:111-112.
[30] Liu, M. et al. Seroprevalence of Toxoplasma gondii infection in pigs in Yunnan. Chinese Livestock Breeding. 12, 111-112 (2011).

[31]洪尼宁,钱德兴,张登祥,华莹,李涛,冉隆仲,田海蓉,刘霞,虞天德.贵州省猪弓形虫病的血清学调查[J].中国动物传染病学报,2010,04:68-70.
[31] Hong, N. et al. Serological survey of porcine toxoplasma infection in Guizhou province. Chinese J. of Animal Infectious Disease. 4, 68-70 (2010).

[32]刘俊伟,姜金庆,李雪华,司红英,谷巧灵.新乡市待宰猪弓形虫感染的血清学调查与分析[J].现代农业科技,2010,18:294+296.
[32] Liu, J., Jiang, J., Li, X., Si, H., Gu, Q. Serological investigation of Toxoplasma gondii infection in slaughter pigs in Xinxiang. Modern Agricultural Science and Technology. 18, 294-296 (2010).

[33]陈永军,王权,蒋蔚,刘迎春,荆振宇,闫叶娜.上海市猪弓形虫流行病学调查[J].中国动物传染病学报,2010,05:54-58.
[33] Chen, Y. et al. Investigation on swine toxoplasmosis in Shanghai suburbs. Chinese J. of Animal Infectious Disease. 5, 54-58 (2010).

[34]董永森,潘英卿,范秀兰,张国仓,刘秀清.青海省猪弓形虫病血清流行病学调查[J].黑龙江畜牧兽医,2010,24:83-84.
[34] Dong, Y., Pan, Y., Fan, X., Zhang, G., Liu, X. Epidemiological investigation of Toxoplasma gondii infection in swine in Qinghai province. Heilongjiang Animal Science and Veterinary Medicine. 24, 83-84 (2010).

[35]姜少敏,陈亚萍,王天奇.伊川县猪弓形虫病血清学调查与分析[J].畜牧兽医杂志,2009,01:35-36.
[35] Jiang, S., Chen, Y., Wang, T. Serological survey and study of pig toxoplasmosis in Yichuan county. J. of Animal Science and Veterinary Medicine. 1, 35-36 (2009).

[36]薛霞,李凯航,邓波,孙泉云.上海地区规模化猪场弓形虫病的血清学调查[J].动物医学进展,2009,03:28-31.
[36] Xue, X., Li, K., Deng, B., Sun, Q. Serological survey on Toxoplasma gondii infection in pig farms in Shanghai. Progress in Veterinary Medicine. 3, 28-31 (2009).

[37]郑华,翟少钦,周雪,徐登峰.荣昌地区猪弓形虫病的血清学调查[J].上海畜牧兽医通讯,2009,06:39-40.
[37] Zeng, H., Qu, S., Zhou, X., Xu, D. Serological survey on Toxoplasma gondii infection in pigs in Rongchang area. Shanghai J. of Animal Husbandry and Veterinary Medicine. 6, 39-40 (2009).

[38]张云峰,刘福元,徐雪萍,何立雄,李爱巧,张雪松.新疆北疆地区弓形虫病的血清学调查[J].草食家畜,2009,04:22-24.
[38] Zhang, Y. et al. Serological survey of Toxoplasma gondii infection in north of Xinjiang. Grass feeding Livestock. 4, 22-24 (2009).

[39]林振营,王天芳,周平,罗才庆,丘寿胜,张华全.龙岩地区部分猪场弓形虫病的血清学调查[J].福建畜牧兽医,2008,03:7-8.
[39] Lin, Z. et al. Serological investigation of swine toxoplasmosis in part farms of Longyan area. Fujian J. of Animal Husbandry and Veterinary Medicine. 3, 7-8 (2008).

[40]谭其军,李艳,聂奎,曾政,杨泽林.重庆地区猪弓形虫血清流行病学调查[J].动物医学进展,2008,07:109-110+115.
[40] Tan, Q., Li, Y., Nie, Q., Zeng, Z., Yang, Z. Serological survey of Toxoplasma gondii infection in Congqing area. Progress in Veterinary Medicine. 7, 110-115 (2008).

[41]王海燕,任庆娥,菅复春,周洋,宁长申,张龙现.河南省部分地区猪弓形虫感染调查及猪源虫株对小鼠的接种试验[J].中国人兽共患病学报,2008,10:930-932.
[41] Wang, H. et al. Investigation on the swine Toxoplasma gondii infection in some districts of Henan province and the inoculation experiment in mice infected with isolates from pigs. Chinese J. of Zoonoses. 10, 930-932 (2008).

[42]米晓云,巴音查汗,李文超.新疆猪、牛、羊弓形虫病的血清学调查[J].中国兽医寄生虫病,2007,02:22-24.
[42] Mi, X., Ba, Y., Li, W. Epidemic investigation of Toxoplasma infection in pigs, cattle and sheep in Xinjiang. Chinese J. of Veterinary Parasitology. 2, 22-24 (2007).

[43]江涛,何会时,赵年彪,韩有元,赵俊龙.湖北省部分猪场弓形虫感染的血清学调查[J].湖北畜牧兽医,2007,06:22+25.
[43] Jiang, T., He, H., Zhao, N., Han, Y., Zhao, J. Serological survey of Toxoplasma gondii infection in some pig farms in Hubei. Hubei J. of Animal and Veterinary Science. 6, 22-25 (2007).

[44]张险朋,李玉娥,温清萍,赖笑娴.东莞市 5 种猪病的血清学调查[J].动物医学进展,2006,01:111-112.
[44] Zhang, X., Li, Y., Wen, Q., Lai, X. Serological study on 5 diseases in pigs in Dongguan. Progress in Veterinary Medicine. 1, 111-112 (2006).

[45]张能贵,刘庆龙,范永婵,张招连,张欣荣.上杭县猪弓形体病的血清学调查[J].福建畜牧兽医,2006,01:34.
[45] Zhang, N., Liu, Q., Fan, Y., Zhang, Z., Zhang, X. Serological investigation on Toxoplasma gondii infection in pigs in Shanghang. Fujian J. of Animal Husbandry and Veterinary Medicine. 1, 34 (2006).

[46]朱继斌.河北省猪弓形虫感染情况调查[J].河北北方学院学报(自然科学版),2006,02:46-48.
[46] Zhu, J. Infection investigation of porcine toxoplasmosis in Hebei. J. of Heibei North University (Natural Science Edition). 2, 46-48 (2006).

[47]刘伯庶,王琼秋,李永斌,郭建新,刘佳升,周小伍.红河州猪弓形虫病血清学调查[J].云南畜牧兽医,2006,04:12-13.
[47] Liu, B. et al. Serological investigation on Toxoplasma gondii infection in pigs in Honghe. Yunnan J. of Animal Husbandry and Veterinary Medicine. 4, 12-13 (2006).

[48]王天奇,董发明,潘耀谦,张玲,方佳培.洛阳地区猪弓形虫病血清学调查与分析[J].湖北畜牧兽医,2006,09:29-30.
[48] Wang, T., Dong, F., Pan, Y., Zhang, L., Fang, J. Serological investigation on Toxoplasma gondii infection in pigs in Luoyang area. Hubei J. of Animal and Veterinary Medicine. 9, 29-30 (2006).

[49]索绪峰,刘建杰,王大林,顾贫,李碧思,王美燕,吴连玉.海南省猪场弓形虫病血清流行病学调查[J].养猪,2006,05:36-37.
[49] Suo, X. et al. Serological investigation on Toxoplasma gondii infection in pigs in Hainan pig farms. Swine Production. 5, 29-30 (2006).

[50]高存福,康桂英,秦建华,顾小龙.河北省猪、奶牛弓形虫病的血清抗体检测[J].中国动物检疫,2005,09:42.
[50] Gao, C., Kang, G., Qin, J., Gu, X. Detection of Toxoplasma gondii infection in pigs and dairy cattle in Hebei province. China Animal Health Inspection. 9, 42 (2005).

[51]王承民,何宏轩,秦建华,姚四新,王丽荣,刘丽艳,牛朝锋,高明超.河南省新乡市郊区猪羊弓形虫病流行病学调查[J].中国寄生虫学与寄生虫病杂志,2005,01:33.
[51] Wang, C. et al. Serological investigation on Toxoplasma gondii infection in pigs and small ruminants in suburbs area in Xinxiang, Henan province. Chin. J. Parasit. Dis. 1, 33 (2005).

[52]苑文英,马凯,刘春颖.河北省动物弓形虫感染情况调查[J].医学动物防制,2004,02:80-82.
[52] Fan, W., Ma, K., Liu, C. Seroprevalence of Toxoplasma gondii infection in animals in Hebei province. Chinese J. of Pest Control. 2, 80-82 (2004).

[53]叶扣贯,范锋,崔洪平,周庆新.无锡地区商品屠宰猪弓形虫感染血清学调查[J].畜禽业,2004,05:43.
[53] Ye, K., Fan, F., Cui, H., Zhou, Q. Seroprevalence of Toxoplasma gondii infection in slaughtered pigs in Wuxi area. Livestock and Poultry Industry. 5, 43 (2004).

[54]傅义娟.青海省猪牛羊弓形虫病的血清抗体检测[J].中国兽医科技,2003,09:69-70.
[54] Fu, Y. Detection of Toxoplasma gondii antibodies in pigs, cattle and small ruminants in Qinghai. Chinese J. of Veterinary and Technology. 9, 69-70 (2003).

[55]孙照学,陈正富,陈永惠,陶迁坤.六枝特区五种畜禽寄生虫感染情况初步调查[J].中国兽医寄生虫病,2003,01:35-37.
[55] Sun, Z., Chen, Z., Chen, Y., Tao, Q. Parasites infection status of 5 different animals in Liuzhi. Chinese J. of Veterinary Parasitology. 1, 35-37 (2003).

[56]阮正祥,王勤,曾志明,付翔,何虎,李竹,杨延军,张强,李存杰,胡永柱,吴建辉,龙厚怡,杨立强,符志明,邹茂华.贵州省毕节地区猪寄生虫区系调查[J].中国兽医寄生虫病,2002,03:16-18.
[56] Ruan, Z. et al. Investigation of parasites infection in pigs in Bijie, Guizhou. Chinese J. of Veterinery Parasitology. 3, 16-18 (2002).

[57]韩秀敏.西宁市商品猪人兽共患寄生虫病感染情况调查[J].青海畜牧兽医杂志,2001,02:21-22.
[57] Han, X. Investigation of zoonotic parasites infection in pigs in Xining. Chinese Qinghai J. of Animal and Veterinary Sciences. 2, 21-22 (2001).

[58]曹兴萍.猪牛弓形虫病的血清学调查[J].当代畜牧,2000,03:21.

[58] Cao, X. Serological survey on Toxoplasma gondii infection in pigs and cattle. Modern Animal Husbandry. 3, 21 (2000).

[59]于三科,林青,刘永庆,王旭宇.陕西杨陵地区猪弓形虫病的血清学调查研究[J].动物医学进展,2000,02:57-59.
[59] Yu, S., Lin, Q., Liu, Y., Wang, X. Serological survey on Toxoplasma gondii infection in pigs in Yangling, Shanxi. Progress in Veterinary Medicine. 2, 57-59 (2000).

[60]王正松,聂浩,方瑞,肖犟,冯辉辉,涂攀,周艳琴,赵俊龙.湖北省规模化猪场弓形虫病动态流行病学调查[A].中国畜牧兽医学会家畜寄生虫学分会.中国畜牧兽医学会家畜寄生虫学分会第六次代表大会暨第十次学术研讨会论文集[C].中国畜牧兽医学会家畜寄生虫学分会:,2009:4.
[60] Wang, Z. et al. Epidemiological survey on Toxoplasma gondii infection in pig farms in Hubei. China Veterinary Medicine Association , 4, (2009).

[61]李金娜.新乡地区动物弓形虫感染情况及危险因素分析[D].新乡医学院,2015.
[61] Li, J. Seroprevalence and risk factor of Toxoplasma gondii infection in animals in Xinxiang. Xinxiang Medicine College. 2015.

[62]穆桂玲.延庆县猪弓形虫病的诊断与流行病学调查[D].中国农业科学院,2009.
[62] Mu, G. Detection and epidemiology survey on Toxoplasma gondii infection in pigs in Yanqin. Chinese Academy of Agriculture Sciences. 2009.

[63]张步彩. 泰州地区猪弓形虫血清流行病学调查及中药治疗急性感染弓形虫小鼠效果的研究[D].扬州大学,2015.
[63] Zhang, B. Seroepidemiological survey on Toxoplasma gondii infection in pigs in Taizhou and effect of Chinese traditional medicine in treating acute infection in mice. Yangzhou University. 2009.

[64] Xu, P. *et al.* Seroprevalence of Toxoplasma gondii infection in pigs in Jilin Province, Northeastern China. *Trop* **32**, 116-120 (2015).

[65] Li, Y. N. *et al.* Seroprevalence and genotype of Toxoplasma gondii in pigs, dogs and cats from Guizhou province, Southwest China. *Parasites & Vectors* **8**, 214, doi:http://dx.doi.org/10.1186/s13071-015-0809-2 (2015).

[66] Jiang, H. H. *et al.* Seroprevalence of Toxoplasma gondii infection in pigs in Jiangxi Province, Southeastern China. *Foodborne Pathog Dis* **11**, 362-365, doi:http://dx.doi.org/10.1089/fpd.2013.1686 (2014).

[67] Chang, Q. C., Zheng, X., Qiu, J. H., Wang, C. R. & Zhu, X. Q. Seroprevalence of Toxoplasma gondii infection in fattening pigs in Northeast China. *Journal of Parasitology* **99**, 544-545, doi:http://dx.doi.org/10.1645/12-102.1 (2013).

[68] Wu, D. *et al.* Seroprevalence of Toxoplasma gondii antibodies from slaughter pigs in Chongqing, China. *Trop Anim Health Prod* **44**, 685-687, doi:http://dx.doi.org/10.1007/s11250-011-9965-3 (2012).

[69] Liu, X. *et al.* Seroprevalence of Toxoplasma gondii infection in slaughtered pigs and cattle in Liaoning Province, northeastern China. *Journal of Parasitology* **98**, 440-441, doi:http://dx.doi.org/10.1645/GE-2989.1 (2012).

[70] Du, F. *et al.* Soil contamination of Toxoplasma gondii oocysts in pig farms in central China. *Veterinary Parasitology* **187**, 53-56, doi:http://dx.doi.org/10.1016/j.vetpar.2011.12.036 (2012).

[71] Yu, H. J. *et al.* Seroprevalence of Toxoplasma gondii infection in pigs, in Zhejiang Province, China. *Journal of Parasitology* **97**, 748-749, doi:http://dx.doi.org/10.1645/GE-2713.1 (2011).

[72] Tao, Q. *et al.* Seroprevalence and risk factors for Toxoplasma gondii infection on pig farms in central China. *Journal of Parasitology* **97**, 262-264, doi:http://dx.doi.org/10.1645/GE-2646.1 (2011).

[73] Zhou, D. H. *et al.* Seroprevalence of Toxoplasma gondii in pigs from southern China. *Journal of Parasitology* **96**, 673-674, doi:http://dx.doi.org/10.1645/GE-2416.1 (2010).

[74] Huang, C. Q. *et al.* Seroprevalence of Toxoplasma gondii infection in breeding sows in Western Fujian Province, China. *Trop Anim Health Prod* **42**, 115-118, doi:http://dx.doi.org/10.1007/s11250-009-9393-9 (2010).

[75] Zou, F. C. *et al.* Seroprevalence of Toxoplasma gondii in pigs in southwestern China. *Parasitology International* **58**, 306-307, doi:http://dx.doi.org/10.1016/j.parint.2009.06.002 (2009).

[76] Shu, F. F. *et al.* Seroprevalence of Toxoplasma gondii Infection in Slaughter Pigs in Sichuan, China. *Journal of Animal and Veterinary Advances* **10**, 1638-1639, doi:10.3923/javaa.2011.1638.1639 (2011).

[77] Wang, D. *et al.* Seroprevalence and genotypes of Toxoplasma gondii isolated from pigs intended for human consumption in Liaoning province, northeastern China. *Parasites and Vectors* **9**, doi:10.1186/s13071-016-1525-2 (2016).

[78] Wu, S. M. *et al.* First report of Toxoplasma gondii prevalence in Tibetan pigs in Tibet, China. *Vector-Borne and Zoonotic Diseases* **12**, 654-656, doi:10.1089/vbz.2012.0968 (2012).

[79] Xu, Y. *et al.* Seroprevalence of toxoplasma gondii infection in sows in hunan province, China. *The Scientific World Journal* **2014**, doi:10.1155/2014/347908 (2014).

[80] Wu, F. *et al.* Seroprevalence and Risk Factors of Toxoplasma gondii in Slaughter Pigs in Shaanxi Province, Northwestern China. *Vector-Borne and Zoonotic Diseases* **17**, 517-519, doi:http://dx.doi.org/10.1089/vbz.2016.210310.1089/vbz.2016.2103

Selected papers for cattle in mainland of China

[1]赵鹏,张守发,贾立军,于龙政.吉林省长春地区牛弓形虫病流行病学调查[A].中国畜牧兽医学会兽医寄生虫学分会.中国畜牧兽医学会兽医寄生虫学分会第十三次学术研讨会论文集[C].中国畜牧兽医学会兽医寄生虫学分会:,2015:1.
[1] Zhao, P., Zhang, S., Jia, L., Yu, L. Epidemiological survey on Toxoplasma gondii infection in cattle in Changchun, Jilin. Animal Husbandry & Veterinary Medicine. 1, (2015).

[2]刘文韬,路义鑫,鹿凌岩,穆永才,刘俊超,戴丽梅,宋铭忻.黑龙江省部分地区牛弓形虫病血清学调查[A].中国畜牧兽医学会家畜寄生虫学分会.中国畜牧兽医学会家畜寄生虫学分会第六次代表大会暨第十次学术研讨会论文集[C].中国畜牧兽医学会家畜寄生虫学分会:,2009:2.

[2] Liu, W. et al. Serological survey on Toxoplasma gondii infection in cattle in Heilongjiang area. Animal Husbandry & Veterinary Medicine. 2, (2009).

[3]蔡进忠,李春花.青海省家畜弓形虫病血清学调查与流行情况分析[A].ChinaSocietyofParasitology、ChinaAssociationforScienceandTechnology.中国动物学会寄生虫学专业委员会第十二次全国学术会议暨第三次国际寄生虫学学术研讨会论文摘要集[C].ChinaSocietyofParasitology、ChinaAssociationforScienceandTechnology:,2009:1.
[3] Cai, J., Li, C. Serological survey on Toxoplasma gondii infection in livestock in Qinghai province. China Society of Parasitology. 1, (2009).

[4]李金娜.新乡地区动物弓形虫感染情况及危险因素分析[D].新乡医学院,2015.
[4] Li, J. Toxoplasma gondii infection and risk factor analysis in animals in Xinxiang area. Xinxiang College. (2015).

[5]崔平.河北省动物弓形虫病流行病学调查[D].河北农业大学,2003.
[5]Cui, P. Epidemiological survey on Toxoplasma gondii infection in animals in Hebei province. Hebei agriculture University. (2003).

[6]何彦春,李珊,李旭蓉,钱振波.甘肃省张掖市动物弓形虫病血清学调查[J].中国动物检疫,2016,v.33;No.27403:12-13.
[6] He, Y., Li, S., Li, X., Qian, Z. Serological survey of animal toxoplasmosis in Zhangye city of Gansu province. China Animal Health Inspection. 33, 12-13 (2016).

[7]董保豫,邓猛,黎桂云,吴永银,倪兴维,王家明,覃倩.黔南州家畜弓形虫病血清学调查[J].黑龙江畜牧兽医,2015,No.48416:94-95.
[7] Dong, B. et al. Serological survey on Toxoplasma gondii infection in livestock in Qiannan. Heilongjiang Animal Science and Veterinary Medicine. 16, 94-95 (2015).

[8]王萌,殷宏,王淑芬,王树清,马玉芬,张德林.甘肃天祝地区牦牛弓形虫病流行病学调查[J].中国奶牛,2015,No.30622:23-25.
[8] Wang, M. et al. Epidemiological survey on Toxoplasma gondii infection in yaks in Tianzhu, Gansu. Dairy Health. 22, 23-25 (2015).

[9]刘辉兰.武平县猪、牛、羊弓形虫病的血清学调查[J].福建畜牧兽医,2014,v.36;No.18602:16-18.
[9] Liu, H. Serological survey on Toxoplasma gondii infection in pigs, cattle and small ruminants in Wupin. Fujian J. of Animal Husbandry and Veterinary Medicine. 36, 16-18 (2014).

[10]张洪波,阿树鹏,仓娘盖,侯红梅,周毛,卡多措,孙亚丽,康明.青海省泽库县牛羊弓形虫病血清学调查[J].畜牧与兽医,2014,v.46;No.34908:125-126.
[10] Zhang, H. et al. Serological survey on Toxoplasma gondii infection in cattle and small ruminants in Zeku, Qinghai province. Animal Husbandry & Veterinary Medicine. 46, 125-126 (2014).

[11]周昕薛,周欢,宁晓冬,李静,菅复春,张龙现,赵青玉,宁长申.我国部分地区牛羊弓形虫血清流行病学调查[J].中国草食动物科学,2014,v.34;No.23505:43-46.
[11] Zhou, X. et al. Serological survey on Toxoplasma gondii infection in cattle, sheep and goats in partial area of China. China Herbivores. 34, 43-46 (2014).

[12]罗才庆,袁匀,黄剑梅,黄翠琴.龙岩市部分地区牛、羊弓形虫病的血清学调查[J].中国动物保健,2013,v.15;No.17307:13-16.
[12] Luo, C., Yuan, J., Huang, J., Huang, C. Epidemic investigation of Toxoplasma infection in cattle and sheep in part of Longyan city. Zhongguo Dong Wu Bao Jian. 15, 13-16 (2013).

[13]杨炬,宋玲,张敏,宋晓佳,马学平,马丽瑶,李丽.宁夏红寺堡区农村居民及家畜弓形虫感染现况调查[J].宁夏医科大学学报, 2012, 34, 46-48.
[13] Yang, J. et al. Survey of Toxoplasma gondii infection in human and livestock in Hongsibao area, Ningxia. J. of Ningxia Medical University. 34, 46-48 (2012).

[14]陆艳,王戈平,蔡其刚,叶成玉,牛小迎,马利青.青海省大通种牛场牦牛弓形虫病的血清学调查[J].中国动物检疫,2012,v.29;No.22603:40-41.
[14] Lu, Y., Serological survey on Toxoplasma gondii infection in yaks in Datong, Qinghai province. China Animal Health Inspection. 29, 40-41 (2012).

[15]孔猛,白昀,丁志勇,刘茂军,冯志新,熊祺琰,白方方,车巧林,王海燕,王占伟,韦艳娜,邵国青.江苏省部分地区动物弓形虫病血清学调查[J].江苏农业科学,2012,v.40, -173.
[15] Kong, M. et al. Serological survey on Toxoplasma gondii infection in animals in Jiangsu area. Jiangsu Agriculture Scinence. 40, 171-173 (2012).

[16]王为升,张金生,陈伟,王俊伟,孟庆玲,乔军.石河子地区人畜弓形虫感染的血清学调查[J].动物医学进展,2011,v.32;No.21202:120-122.
[16] Wang, W. et al. Serological survey on Toxoplasma gondii infection in human and livestock in Shihezi area. Progress in Veterinary Medicine. 32, 120-122 (2011).

[17]董永森,罗自清,张国仓,刘秀清.青海省牛羊弓形虫病流行病学调查[J].中国人兽共患病学报,2011,v.2704:359+363.
[17] Dong, Y., Luo, Z., Zhang, G., Liu, X. Epidemiological survey on Toxoplasma gondii infection in cattle and small ruminant in Qinghai province. Chinese J. of Zoonoses. 2704, 359 (2011).

[18]赵全邦,胡广卫,李静,李连芳,马占全,袁友贞,陈辅君,潘雪英.青海省德令哈地区牛弓形虫病血清学调查[J].畜牧与兽医,2011,v.43;No.30904:103-104.
[18] Zhao, Q. et al. Serological survey on Toxoplasma gondii infection in cattle and small ruminant in Delinha area, Qinghai province. Animal Husbandry and Veterinary Medicine. 43, 103-104 (2011).

[19]李建萍.西山区 5 种动物血清弓形虫抗体调查研究[J].中外医疗,2011,v.3024:125.
[19] Li, J. Study on Toxoplasma gondii antibodies in 5 animals in Xishan district. China Foreign Medical Treatment. 3024, 125 (2011).

[20]李英,李增魁,圈华,李永坚,侯凯.青海省互助县牦牛弓形虫病血清学调查[J].中国动物检疫,2010,v.27;No.20304:52-53.
[20] Li, Y., Li, Z., Quan, H., Li, Y., Hou, K. Serological survey of yak toxoplasmosis in Huzhu county. Qinghai province. China Animal Health Inspection. 27, 52-53 (2010).

[21]张云峰,刘福元,徐雪萍,何立雄,李爱巧,张雪松.新疆北疆地区弓形虫病的血清学调查[J].草食家畜,2009,No.14504:22-24.
[21] Zhang, Y. et al. Serological survey on Toxoplasma gondii infection in north Xinjiang. Grass Feeding Livestock. 14504, 22-24 (2009).

[22]米晓云,巴音查汗,李文超.新疆猪、牛、羊弓形虫病的血清学调查[J].中国兽医寄生虫病,2007,No.5802:22-24.
[22] Mi, X., Ba, Y., Li, W. Epidemic investigation of Toxoplasma infection in pigs, cattle and sheep in Xinjiang. Chinese Journal of Veterinary Parasitology. 5802, 22-24 (2007).

[23]苑文英,马凯,刘春颖.河北省动物弓形虫感染情况调查[J].医学动物防制,2004,02:80-82.
[23] Fan, W., Ma, K, Liu, C. Survey on Toxoplasma gondii infection in animals in Hebei. J. of Medical Pest Control. 2, 80-82 (2004).

[24]崔平,方素芳,武治云,武保青.河北省家畜弓形虫病的流行病学调查[J].中国兽医科技,2004,11:35-36.
[24] Cui, P., Fang, S., Wu, Z., Wu, B. Epidemiological survey on Toxoplasma gondii infection in livestock in Hebei province. 11, 35-36 (2004).

[25]傅义娟.青海省猪牛羊弓形虫病的血清抗体检测[J].中国兽医科技,2003,09:69-70.
[25] Fu, Y. Detection of Toxoplasma gondii infection in pigs, cattle and small ruminant in Qinghai province. Chinese J. of Veterinary Science and Technology. 9, 69-70 (2003).

[26]白万胜,陈瑛,巴垃提,买买提.阿克苏地区家畜弓形虫病的血清学调查[J].中国兽医寄生虫病,2002,02:29-30.
[26] Bai, W., Chen, Y., Ba, L., Mai, M. Serological survey on Toxoplasma gondii infection in livestock in Akesu area. Chinese J. of Veterinary Parasitology. 2, 29-30 (2002).

[27]曹兴萍.猪牛弓形虫病的血清学调查[J].当代畜牧,2000,03:21.
[27] Cao, X. Serological survey on Toxoplasma gondii infection in pigs and cattle. Modern Animal Husbandry. 3, 21 (2000).

[28]张子群,杨凤新,金宁,耿炳华.牛弓形虫的血清学调查[J].肉品卫生,2000,01:14.
[28] Zhang, Z. Yang, F., Jin, N. Gen, B. Serological survey on Toxoplasma gondii infection in cattle. Meat Hygiene. 1, 14 (2000).

[29] Wang, M. *et al.* Serological survey of Toxoplasma gondii in Tibetan mastiffs (Canis lupus familiaris) and yaks (Bos grunniens) in Qinghai, China. *Parasit Vectors* **5**, 35, doi:http://dx.doi.org/10.1186/1756-3305-5-35 (2012).

[30] Ge, W. *et al.* Prevalence and genotype of Toxoplasma gondii infection in cattle from Jilin Province, northeastern China. *Vector Borne & Zoonotic Diseases* **14**, 399-402, doi:http://dx.doi.org/10.1089/vbz.2013.1516 (2014).

[31] Qiu, J. H. *et al.* Seroprevalence of Toxoplasma gondii in beef cattle and dairy cattle in northeast China. *Foodborne Pathog Dis* **9**, 579-582, doi:http://dx.doi.org/10.1089/fpd.2011.1104 (2012).

[32] Liu, X. *et al.* Seroprevalence of Toxoplasma gondii infection in slaughtered pigs and cattle in Liaoning Province, northeastern China. *Journal of Parasitology* **98**, 440-441, doi:http://dx.doi.org/10.1645/GE-2989.1 (2012).

[33] Liu, Q. *et al.* Seroprevalence of Toxoplasma gondii infection in yaks (Bos grunniens) in northwestern China. *Trop Anim Health Prod* **43**, 741-743, doi:http://dx.doi.org/10.1007/s11250-010-9711-2 (2011).

[34] Liu, J. *et al.* Seroepidemiology of Neospora caninum and Toxoplasma gondii infection in yaks (Bos grunniens) in Qinghai, China. *Veterinary Parasitology* **152**, 330-332, doi:http://dx.doi.org/10.1016/j.vetpar.2007.12.010 (2008).

[35] Li, K. *et al.* Seroprevalence of Toxoplasma gondii infection in yaks (Bos grunniens) on the Qinghai-Tibetan Plateau of China. *Veterinary parasitology* **205**, 354-356, doi:10.1016/j.vetpar.2014.07.014 (2014).

[36] Qin, S. Y. *et al.* Seroprevalence, risk factors and genetic characterization of Toxoplasma gondii in free-range white yaks (Bos grunniens) in China. *Veterinary parasitology* **211**, 300-302, doi:10.1016/j.vetpar.2015.05.015 (2015).

[37] Luo, H. *et al.* Seroprevalence of Toxoplasma gondii infection in zoo and domestic animals in Jiangxi Province, China. *Parasite (Paris, France)* **24**, 7, doi:http://dx.doi.org/10.1051/parasite/201700710.1051/parasite/2017007

[38] Yu, J. *et al.* Seroepidemiology of Neospora caninum and Toxoplasma gondii in cattle and water buffaloes (Bubalus bubalis) in the People's Republic of China. *Vet Parasitol* **143**, 79-85 (2007).

Selected papers for chicken in mainland of China

[1]李金娜.新乡地区动物弓形虫感染情况及危险因素分析[D].新乡医学院,2015.
[1] Li, J. Toxoplasma gondii infection in animals and risk factor analysis in Xinxiang. Xinxiang Medical College. 2015.

[2]刘欣超.我国部分地区鸡肉、羊肉弓形虫感染及土壤污染情况调查[D].南京农业大学,2013.
[2] Liu, X. Toxoplasma gondii infection in chicken and small ruminant meats and soil contamination in China. Nanjing Agriculture University. 2013

[3]张梦.我国部分地区鸡、猪及几种淡水生物弓形虫感染情况调查[D].南京农业大学,2012.
[3] Zhang, M. Survey on Toxoplasma gondii infection in chicken, pigs and animals in freshwater in China. Nanjing Agriculture University. 2012.

[4]龙祥.荆州市鸡弓形虫病血清学调查[D].长江大学,2013.
[4] Long, X. Serological investigation of Toxoplasma gondii infection in Chicken in Jingzhou. Changjiang University. 2013.

[5]王凤阳.弓形虫改良凝集试验的建立及我国部分地区犬、鸡弓形虫病流行病学调查[D].吉林农业大学,2012.
[5] Wang, F. Development of Toxoplasma gondii MAT test and epidemiological survey of Toxoplasma gondii infection in dogs and chicken in China.

[6]何彦春,李珊,李旭蓉,钱振波.甘肃省张掖市动物弓形虫病血清学调查[J].中国动物检疫,2016,03:12-13.
[6] he, Y. Li, S. Li, X. Qian, Z. Serological Survey of animal Toxoplasmosis in Zhangye city of Gansu province. China Animal Health Inspection. 3, 12-13 (2016).

[7]王大为,韩小虎,慕名扬,袁高明,张国新,何剑斌,杨娜,李红魁.中国东北地区部分动物弓形虫病的流行病学调查[J].黑龙江畜牧兽医,2014,23:129-131.
[7] Wang, D. et al. Epideiological survey of toxoplasmosis in some animals from northeastern China. Heilongjiang Animal Science and Veterinary Medicine. 23, 129-131 (2014).

[8]雷程红,蔡元庆,包振中,卞赛赛,高窦.散养鸡场中散养鸡和麻雀弓形虫感染情况的血清学调查[J].黑龙江畜牧兽医,2015,04:71-72.
[8] Lei, C. Cai, Y., Bao, Z., Bian,S., Gao, D. Serological investigation of Toxoplasma gondii infection of free-range chickens and sparrows in a free-range chicken farm. Heilongjiang Animal Science and Veterinary Medicine. 4, 71-72 (2015).

[9]朱骏,沈明华,陈露儿,曹向英,孙文梅,金一春.松江区家禽弓形虫感染的血清学调查[J].上海畜牧兽医通讯,2015,06:32-33.
[9] Zhu, J. et al. Serological survey on Toxoplasma gondii infection in poultry in Songjiang district. Shanghai J. of Animal Science and Veterinary Medicine. 6, 32-33 (2015).

[10]许越,王凤阳,刘贤英,魏峰,刘全.改良凝集试验(MAT)检测鸡弓形虫血清抗体[J].中国兽医学报,2014,11:1781-1782+1789.
[10] Xu, Y., Wang, F., Liu, X., Wei, F., Liu, Q. A modified agglutination test for diagnosing toxoplasmosis in chicken. Chin. J. Vet. Sci. 11, 1781-1782 (2014).

[11]雷程红,郭芙莉,王伊丽,包振中,蔡元庆.乌鲁木齐地区猪犬鸡弓形虫病血清学调查[J].新疆畜牧业,2013,11:28-30.
[11] Lei, C., Guo, F., Wang, Y., Bao, Z., Cai, Y. Serological investigation of Toxoplasma gondii infection in pigs, dogs and chicken in Wulumuqi. Xinjiang Xu Mu Ye. 11, 28-30 (2013).

[12]刘容珍,陈志清,张创峰,钟万朝,陈明霞.广东部分地区鸡弓形虫感染的血清学调查[J].养禽与禽病防治,2013,03:7-9.
[12] Liu, R., Chen, Z., Zhang, C., Zhong, W., Chen, M. Serological survey on Toxoplasma gondii infection in chicken in Guangdong area. Yang Qin Yu Qin Bin Fang Zhi. 3, 7-9 (2013).

[13]苑文英,马凯,杨宏莉.河北省动物弓形虫感染调查[J].中国血吸虫病防治杂志,2004,01:72-76.
[13] Fan, W., Ma, K., Yang, H. Toxoplasma gondii infection in animals in Hebei. *Chin. J. Schisto. Control.* 1, 72-76 (2004).

[14] Zhao, G. *et al.* Detection of Toxoplasma gondii in free-range chickens in China based on circulating antigens and antibodies. *Veterinary Parasitology* **185**, 72-77, doi:http://dx.doi.org/10.1016/j.vetpar.2011.10.031 (2012).

[15] Cong, W. *et al.* First report of Toxoplasma gondii infection in market-sold adult chickens, ducks and pigeons in northwest China. *Parasites & Vectors [Electronic Resource]* **5**, 110, doi:http://dx.doi.org/10.1186/1756-3305-5-110 (2012).
[16] Yan, C. *et al.* Toxoplasma gondii infection in domestic ducks, free-range and caged chickens in southern China. *Veterinary Parasitology* **165**, 337-340, doi:http://dx.doi.org/10.1016/j.vetpar.2009.07.015 (2009).

[17] Zhu, J. *et al.* A sero-epidemiological survey of Toxoplasma gondii infection in free-range and caged chickens in northeast China. *Veterinary Parasitology* **158**, 360-363, doi:http://dx.doi.org/10.1016/j.vetpar.2008.09.024 (2008).

[18] Feng, Y. *et al.* Toxoplasma gondii and Neospora caninum in Free-Range Chickens in Henan Province of China. *BioMed Research International* **2016**, doi:10.1155/2016/8290536 (2016).
[19] Ma, L., Wang, Z. D., Li, J. P., Wei, F. & Liu, Q. Seroprevalence of toxoplasma gondii infection in freerange chickens in Jilin Province, northeastern China. *Tropical Biomedicine* **32**, 693-698 (2015).
[20] Xu, P. *et al.* Seroprevalence of Toxoplasma gondii infection in chickens in Jinzhou, northeastern China. *J Parasitol* **98**, 1300-1301, doi:http://dx.doi.org/10.1645/GE-3164.1

Selected papers for small ruminant in mainland of China

[1]何彦春,李珊,李旭蓉,钱振波.甘肃省张掖市动物弓形虫病血清学调查[J].中国动物检疫,2016,v.33;No.27403:12-13.
[1] he, Y. Li, S. Li, X. Qian, Z. Serological Survey of animal Toxoplasmosis in Zhangye city of Gansu province. China Animal Health Inspection. 3, 12-13 (2016).

[2]王青青,黄耀杰,张婉琪,孙皓,陈瑛,库尔班妮萨·图尔荪,胡建军.新疆南疆某规模化羊场弓形虫病的血清学调查[J].畜牧与饲料科学,2016,v.37;No.21603:107-109.
[2] Wang, Q. et al. Serological Survey of Toxoplasma gondii infection in small ruminant farms in north Xinjiang. Animal Husbandry and Feed Science. 37, 12-13 (2016).

[3]祖阿丽娅,雷程红,卞赛赛,高窦,葛婷.屠宰羊弓形虫感染情况调查[J].畜牧与饲料科学,2014,v.35;No.20112:96.
[3] Z, A., Lei, C., Bian, S., Gao, D., Ge, T. Investigation on Toxoplasma gondii infection of slaughtered sheep. Animal Husbandry and Feed Science. 35, 96 (2014).

[4]王大为,韩小虎,慕名扬,袁高明,张国新,何剑斌,杨娜,李红魁.中国东北地区部分动物弓形虫病的流行病学调查[J].黑龙江畜牧兽医,2014,No.46723:129-131.
[4] Wang, D. et al. Epidemiological survey of Toxoplasmosis in some animals from northeastern China. Heilongjiang Animal Science and Veterinary Medicine. 46723, 129-131 (2014).

[5]王洁,张中义,周正永,姚人文,熊毅,韦永江,汪达卫,张元富,董宝豫.罗甸县羊寄生虫病调查结果初报[J].农民致富之友,2015,No.50104:142.
[5] Wang, J. et al. Preliminary report on investigation of parasites disease in small ruminants in Luodian county. NonMing ZhiFu ZhiYou. 50104, 142 (2015).

[6]赵良.兴海县藏系绵羊弓形虫病血清学调查[J].中国畜牧兽医文摘,2015,v.31;No.20608:115.
[6] Zhao, L. Serological survey of Toxoplasma gondii infection in Tibetan sheep in Xinhai county. Chinese Abstracts of Animal Husbandry and Veterinary Medicine. 31, 115 (2015).

[7]欧阳仙,徐聪,薛涛,杨耀兰,吕嵘,宋红莲,孙绍萍.玉溪市山羊弓形虫病的血清学调查[J].云南畜牧兽医,2014,No.17003:12-13.
[7] Ouyang, X. et al. Serological survey on Toxoplasma gondii infection in goats in Yuxi. Yunnan J. of Animal Husbandry and Veterinary Medicine. 17003, 12-13 (2014).

[8]张洪波,阿树鹏,仓娘盖,侯红梅,周毛,卡多措,孙亚丽,康明.青海省泽库县牛羊弓形虫病血清学调查[J].畜牧与兽医,2014,v.46;No.34908:125-126.

[8] Zhang, H. et al. Serological investigation on Toxoplasma gondii infection in cattle and small ruminants in Kuze, Qinghai province. 46, 125-126 (2014).

[9]吕望海.共和县藏系羊弓形虫病的血清学调查[J].山东畜牧兽医,2014,v.35;No.21209:65.
[9] Lv, W. Serological survey on Toxoplasma gondii infection in Tibetan sheep in Gonghe county. Shandong J. of Animal Husbandry and Veterinary Medicine. 35, 65 (2014).

[10]董保豫,钱德兴,黎桂云,吴永银,王兴辉,陈波,覃倩.黔南州山羊弓形虫病血清学调查[J].中国兽医杂志,2014,v.5009:33-34.
[10] Dong, B. et al. Serological survey on Toxoplasma gondii infection in goats in Qiannan. Chinese J. of Veterinary Medicine. 5009, 33-34 (2014).

[11]周昕薛,周欢,宁晓冬,李静,菅复春,张龙现,赵青玉,宁长申.我国部分地区牛羊弓形虫血清流行病学调查[J].中国草食动物科学,2014,v.34;No.23505:43-46.
[11] Zhou, X. et al. Seroprevalence of Toxoplasma gondii in cattle, sheep and goats in partial area of China. China Herbivores. 34, 43-46 (2014).

[12]罗才庆,袁匀,黄剑梅,黄翠琴.龙岩市部分地区牛、羊弓形虫病的血清学调查[J].中国动物保健,2013,v.15;No.17307:13-16.
[12] Luo, C., Yuan, Y., Huang, J., Huang, C., Epidemic investigation of Toxoplasma infection in cattle and sheep in part of Longyan city. Zhongguo Dongwu Baojian. 15, 13-16 (2013).

[13]魏赐开,刘兵,范中孝.瓮安县山羊寄生虫病调查[J].湖北畜牧兽医,2013,v.3407:49-50.
[13] Wei, C., Liu, B. Fan, Z. Survey on parasitic disease in goats in Wenan county. 3407, 49-50 (2013).

[14]毛坤明,林曾欢,林秀敏,林智勇,陈勇,吴丹.福清山羊衣原体病、弓形虫病和布鲁氏菌病血清学调查[J].福建畜牧兽医,2013,v.35;No.18305:21-22.
[14] Mao, K. et al. Inestigation of Chlamydia, Toxoplasma gondii and Brucella infection in goats in Fuqinshan. Fujian J. of Animal Husbandry and Veterinary Medicine. 35, 21-22 (2013).

[15]康明,李英,石文辉,任旭荣,张春霞,张丽婕.青海省化隆县牛羊弓形虫病血清学调查[J].畜牧与兽医,2013,v.45;No.33910:128.
[15] Kang, M. et al. Serological investigation on Toxoplasma gondii infection in cattle and small ruminants in Hualong, Qinghai province.

[16]李万财.藏羊的弓形虫衣原体和布鲁菌病的血清抗体检测[J].中国兽医杂志,2012,v.4802:58-59.
[16] Li, W. Detection of Toxoplasma gondii, Chlamydia and Brucella antibodies in Tibetan sheep. Chinese J. of Veterinary Medicine. 4802, 58-59 (2012).

[17]孔猛,白昀,丁志勇,刘茂军,冯志新,熊祺琰,白方方,车巧林,王海燕,王占伟,韦艳娜,邵国青.江苏省部分地区动物弓形虫病血清学调查[J].江苏农业科学,2012,v.40;No.28602:171-173.
[17] Kong, M. et al. Serological survey on Toxoplasma gondii infection in animals in Jiangsu province. Jiangsu Agriculture Sciences. 40, 171-173 (2012).

[18]汪洪冰,范仲鑫,黄建龙,邓国华,刘道新,王昌建,邱立新,鲁杏华,何世成,张菁.2011 年桃江县规模山羊场弓形虫病监测[J].湖南畜牧兽医,2012,No.17206:26-28.
[18] Wang, H. et al. Surveillance of Toxoplasma gondii infection in goat farms in Taojiang in 2011. Hunan Animal Science and Veterinary Medicine. 17206, 26-28 (2012).

[19]蔡进忠,李春花.青海家畜弓形虫病血清学调查与流行情况分析[J].中国兽医杂志,2011,v.4701:45-46.
[19] Cai, J., Li, C. Serological survey and epidemiological analysis on Toxoplasma gondii infection in livestock in Qinghai. Chinese J. of Veterinary Medicine. 4701, 45-46 (2011).

[20]董永森,罗自清,张国仓,刘秀清.青海省牛羊弓形虫病流行病学调查[J].中国人兽共患病学报,2011,v.2704:359+363.
[20] Dong, Y., Luo, Z., Zhang, G., Liu, X. Epidemiological survey on Toxoplasma gondii infection in cattle and small ruminant in Qinghai province. Chinese J. of Zoonoses. 2704, 359-363 (2011).

[21]赵全邦,胡广卫,李静,李连芳,马占全,袁友贞,陈辅君.青海省德令哈地区羊弓形虫病血清学调查[J].上海畜牧兽医通讯,2011,No.17402:51.
[21] Zhao, Q. et al. Serological survey on Toxoplasma gondii infection in small ruminant in Delinha area, Qinghai province. Shanghai J. of Animal Husbandry and Veterinary Medicine. 17402, 51 (2011).

[22]李建萍.西山区 5 种动物血清弓形虫抗体调查研究[J].中外医疗,2011,v.3024:125.
[22] Li, J. Toxoplasma gondii antibodies detection in 5 different animals in Xishan district. China Foreign Medical Treatment. 3024, 125 (2011).

[23]李永光.青海省民和县陶塞特羊及其后代羊弓形虫病的血清学调查[J].畜牧与饲料科学,2011,v.32;No.16411:108.
[23] Li, Y. Serological survey on Toxoplasma gondii infection in Taosaite sheep and their offspring in Minghe, Qinghai province. Animal Husbandry and Feed Science. 32, 108 (2011).

[24]李英,李增魁,康明,李永坚,刘李盛.青海省互助县藏羊弓形虫血清学调查[J].动物医学进展,2010,v.31;No.20507:119-121.
[24] Li, Y., Li, Z., Kang, M. Li, Y., Liu, L., Serological survey on Toxoplasma gondii infection in Tibetan sheep in Huzhu Qinghai province. Progress in Veterinary Medicine. 31, 119-121 (2010).

[25]张晓强,李万财,牛小迎.天峻县土种藏系羊弓形虫病的血清学调查[J].青海畜牧兽医杂志,2009,v.39;No.19901:29.
[25] Zhang, X., Li, W., Niu, X. Serological survey on Toxoplasma gondii infection in Tibetan sheep in Tianjuan county. Chinese Qinghai J. of Animal and Veterinary Sciences. 39, 29 (2009).

[26]贾俊元,高生智,史万贵,张建军.甘肃省规模养羊场羊传染病与寄生虫病的调查[J].中国动物检疫,2009,v.26;No.18902:44-46.
[26] Jia, J., Gao, S., Shi, W., Zhang, J. Investigation of infectious and parasitic diseases in small ruminant farms in Gansu. China Animal Health Inspection. 26, 44-46 (2009).

[27]张晓强,陆艳,李万财.青海省天峻县藏系羊弓形虫病的血清学调查[J].中国动物检疫,2009,v.26;No.19407:44-45.
[27] Zhang, X., Lu, Y., Li, W. Serological survey on Toxoplasma gondii infection in Tibetan sheep in Tianjuan county. China Animal Health Inspection. 26, 44-45 (2009).

[28]张云峰,刘福元,徐雪萍,何立雄,李爱巧,张雪松.新疆北疆地区弓形虫病的血清学调查[J].草食家畜,2009,No.14504:22-24.
[28] Zhang, Y. et al. Serological survey on Toxoplasma gondii infection in north Xinjiang. Grass Feeding Livestock. 14504, 22-24 (2009).

[29]陈才英.大通县家畜弓形虫病流行病学调查[J].青海畜牧兽医杂志,2008,No.19301:23.
[29] Chen, C. Epidemiological survey on Toxoplasma gondii infection in livestock in Datong county. 19301, 23 (2008).

[30]陈亮,窦永喜,田斌,胡正艳,王宗元.兰州地区羊寄生虫感染情况调查及防治策略[J].甘肃畜牧兽医,2008,No.20306:18-20.
[30] Chen, L., Dou, Y., Tian, B., Hu, Z., Wang, Z. Investigation of parasitic disease in small ruminant in Lanzhou and control strategies. Gansu Animal and Veterinary Sciences. 20306, 18-20 (2008).

[31]米晓云,巴音查汗,李文超.新疆猪、牛、羊弓形虫病的血清学调查[J].中国兽医寄生虫病,2007,No.5802:22-24.
[31] Mi, X., Ba, Y., Li, W. Epidemic investigation of Toxoplasma infection in pigs, cattle and sheep in Xinjiang. Chinese J. of Veterinary Parasitology. 5802, 22-24 (2007).

[32]原永海,马利青.青海省引进小尾寒羊及其后代羊弓形虫病的血清学调查[J].畜牧与兽医,2007,No.26609:75.
[32] Yuan, Y., Ma, L. Serological investigation on Toxoplasma gondii infection in Xiaoweihan sheep and their offspring in Qinghai. Animal Husbandry and Veterinary Medicine. 26609, 75 (2007).

[33]苑文英,马凯,杨宏莉.河北省动物弓形虫感染调查[J].中国血吸虫病防治杂志,2004,01:72-76.
[33] Fan, W., Ma, K., Yang, H. Toxoplasma gondii infection in animals in Hebei province, *Chin. J. Schisto. Control* 1, 72-76 (2004).

[34]傅义娟.青海省猪牛羊弓形虫病的血清抗体检测[J].中国兽医科技,2003,09:69-70.
[34] Fu, Y. Serological survey on Toxoplasma gondii infection in pigs, cattle and chicken. Chinese J. of Veterinary Science and Technology. 9, 69-70 (2003).

[35]蔡金山,赵全帮,李连芳,马占全.青海省牛、羊弓形虫病血清学调查[A].中国动物学会寄生虫学专业委员会第十二次全国学术会议暨第三次国际寄生虫学学术研讨会论文摘要集[C]. 2009, 1.
[35] Cai, J., Zhao, Q., Li, L., Ma, Z. Serological survey on Toxoplasma gondii infection in cattle and small ruminant in Qinghai. China Association for Science and Technology. 1 (2009).

[36]杨娜,郝攀,刘功振,赵现龙,王辉,刘群.内蒙古某地绵羊、山羊新孢子虫和弓形虫血清抗体检测初报[A].中国畜牧兽医学会家畜寄生虫学分会.中国畜牧兽医学会家畜寄生虫学分会第六次代表大会暨第十一次学术研讨会论文集[C].中国畜牧兽医学会家畜寄生虫学分会:,2011:2.
[36] Yang, N. et al. Preliminary report of Neospora canunum and Toxoplasma gondii antibodies detection in sheep and goats in Inner Mongolia. China Association for Animal Husbandry and Veterinary Medicine. 2, (2011).

[37]崔平,秦建华,方素芳.河北省动物弓形虫病流行病学调查[A].中国畜牧兽医学会家畜寄生虫学分会.中国畜牧兽医学会家畜寄生虫学分会第五次代表大会暨第八次学术研讨会论文集[C].中国畜牧兽医学会家畜寄生虫学分会:,2004:4.
[37] Cui, P. Qin, J., Fang, S. Epidemiological survey on Toxoplasma gondii infection in Hebei province. China Association for Animal Husbandry and Veterinary Medicine. 4, (2004).

[38] Yin, M. Y. *et al.* Seroprevalence and risk factors of Toxoplasma gondii in Tibetan Sheep in Gansu province, Northwestern China. *BMC Vet Res* **11**, 41, doi:http://dx.doi.org/10.1186/s12917-015-0358-0 (2015).

[39] Yang, N., Li, H., He, J., Mu, M. & Yang, S. Seroprevalence of Toxoplasma gondii infection in domestic sheep in Liaoning Province, northeastern China. *Journal of Parasitology* **99**, 174-175, doi:http://dx.doi.org/10.1645/GE-3201.1 (2013).

[40] Liu, Q. *et al.* Seroprevalence of Toxoplasma gondii infection in Tibetan sheep in northwestern China. *Journal of Parasitology* **96**, 1222-1223, doi:http://dx.doi.org/10.1645/GE-2601.1 (2010).

[41] Li, F. *et al.* Seroprevalence of Toxoplasma gondii in goats in Hunan province, China. *Parasite* **23**, doi:10.1051/parasite/2016053 (2016).

[42] Liu, Z. K., Li, J. Y. & Pan, H. Seroprevalence and risk factors of Toxoplasma gondii and Neospora caninum infections in small ruminants in China. *Preventive veterinary medicine* **118**, 488-492, doi:10.1016/j.prevetmed.2014.12.017 (2015).

[43] Luo, H. Q. *et al.* Seroepidemiology of Toxoplasma gondii and Neospora caninum infections in goats in Hubei Province, China. *Tropical Biomedicine* **33**, 285-289 (2016).

[44] Wang, C. R. *et al.* Seroprevalence of Toxoplasma gondii infection in sheep and goats in northeastern China. *Small Ruminant Research* **97**, 130-133, doi:10.1016/j.smallrumres.2011.02.009 (2011).

[45] Xu, P. *et al.* Seroprevalence and risk factors for Toxoplasma gondii in sheep and goats in Jinzhou, Northeastern China. *Tropical Biomedicine* **32**, 563-567 (2015).

[46] Zhao, G. H. *et al.* Seroprevalence of Toxoplasma gondii infection in dairy goats in Shaanxi Province, Northwestern China. *Parasites and Vectors* **4**, doi:10.1186/1756-3305-4-47 (2011).

[47] Zou, F. *et al.* Seroprevalence and risk factors of toxoplasma gondii infection in buffaloes, sheep and goats in Yunnan province, Southwestern China. *Iranian Journal of Parasitology* **10**, 648-651 (2015).

[48] Yang, Y. *et al.* Seroprevalence, isolation, genotyping, and pathogenicity of Toxoplasma gondii strains from sheep in China. *Frontiers in Microbiology* **8**, doi:http://dx.doi.org/10.3389/fmicb.2017.0013610.3389/fmicb.2017.00136

[49] Luo, H. *et al.* Seroprevalence of Toxoplasma gondii infection in zoo and domestic animals in Jiangxi Province, China. *Parasite (Paris, France)* **24**, 7, doi:http://dx.doi.org/10.1051/parasite/201700710.1051/parasite/2017007

[50] Zhang, N. *et al.* Seroprevalence of Toxoplasma gondii infection and risk factors in domestic sheep in Henan province, central China. *Parasite (Paris, France)* **23**, 53

[51] Wu, S. M. *et al.* Seroprevalence of Toxoplasma gondii infection in Tibetan sheep in Tibet, China. *J Parasitol* **97**, 1188-1189, doi:http://dx.doi.org/10.1645/GE-2912.1 (2011).

Selected papers for duck in mainland of China

[1]王大为,韩小虎,慕名扬,袁高明,张国新,何剑斌,杨娜,李红魁.中国东北地区部分动物弓形虫病的流行病学调查[J].黑龙江畜牧兽医,2014,23:129-131.
[1] Wang, D. et al. Epidemiological survey of toxoplasmosis in some animals from northeastern China. Heilongjiang Animal Science and Veterinary Medicine. 23, 129-131 (2014).

[2]朱骏,沈明华,陈露儿,曹向英,孙文梅,金一春.松江区家禽弓形虫感染的血清学调查[J].上海畜牧兽医通讯,2015,06:32-33.
[2] Zhu, J. et al. Serological survey on Toxoplasma gondii infection in poultry in Songjiang district. Shanghai J. of Animal Husbandry and Veterinary Medicine.

[3] Yan, C. *et al.* Toxoplasma gondii infection in domestic ducks, free-range and caged chickens in southern China. *Veterinary Parasitology* **165**, 337-340, doi:http://dx.doi.org/10.1016/j.vetpar.2009.07.015 (2009).

[4] Zhao, G. *et al.* A seroepidemiological survey of Toxoplasma gondii infection in free-range and caged ducks in Southwest China. *Israel Journal of Veterinary Medicine* **70**, 41-45 (2015).

[5] Cong, W. *et al.* First report of Toxoplasma gondii infection in market-sold adult chickens, ducks and pigeons in northwest China. *Parasit Vectors* **5**, 110, doi:http://dx.doi.org/10.1186/1756-3305-5-110 (2012).

Selected papers for goose in mainland of China

[1]王大为,韩小虎,慕名扬,袁高明,张国新,何剑斌,杨娜,李红魁.中国东北地区部分动物弓形虫病的流行病学调查[J].黑龙江畜牧兽医,2014,23:129-131.
[1] Wang, D. et al. Epidemiological survey of toxoplasmosis in some animals from northeastern China. Heilongjiang Animal Science and Veterinary Medicine. 23, 129-131 (2014).

[2]王新秋,高艳,李功勇,何勇,袁子国,翁亚彪,林瑞庆.广东清远鹅弓形虫病血清学调查[J].中国兽医杂志,2012,03:10-12.
[2] Wang, X., et al. Survey of Toxoplasma gondii in geese from Qingyuan city, Guangdong province. Chinese J. of Veterinary Medicine. 3, 10-12 (2012).

[3] Rong, G. *et al.* Seroprevalence, risk factors and genotyping of Toxoplasma gondii in domestic geese (Anser domestica) in tropical China. *Parasites & Vectors [Electronic Resource]* **7**, 459, doi:http://dx.doi.org/10.1186/s13071-014-0459-9 (2014).

[4] Yan, C. *et al.* Serological survey of Toxoplasma gondii infection in the domestic goose (Anser domestica) in southern China. *Zoonoses Public Health* **58**, 299-302, doi:http://dx.doi.org/10.1111/j.1863-2378.2010.01349.x (2011).

[5] Tan, S. G., Zhang, M. J., Liu, N. & Xu, P. Y. First report of seroprevalence of Toxoplasma gondii in domestic geese in hunan province, subtropical China. *Tropical Biomedicine* **33**, 366-369 (2016).

Selected papers for donkey in mainland of China

[1]苑文英,马凯,刘春颖.河北省动物弓形虫感染情况调查[J].医学动物防制,2004,02:80-82.

[1] Fan, W., Ma, K., Yang, H. Toxoplasma gondii infection in animals in Hebei province, *Chin. J. Schisto. Control* 1, 72-76 (2004).

[2]崔平,方素芳,武治云,武保青.河北省家畜弓形虫病的流行病学调查[J].中国兽医科技,2004,11:35-36.
[2] Cui, P., Fang, S., Wu, Z., Wu, B. Epidemiological survey on Toxoplasma gondii infection in livestock in Hebei province. Chinese J. of Veterinary Medicine and Technology. 11, 35-36 (2004).

[3]白万胜,陈瑛,巴垃提,买买提.阿克苏地区家畜弓形虫病的血清学调查[J].中国兽医寄生虫病,2002,02:29-30.
[3] Bai, W., Chen, Y., Ba, L., Mai, M. Serological survey on Toxoplasma gondii infection in livestock in Akesu area. Chinese J. of Veterinary Parasitology. 2, 29-30 (2002).

[4] Yang, N. *et al.* Seroprevalence of Toxoplasma gondii in slaughtered horses and donkeys in Liaoning province, northeastern China. *Parasites & Vectors [Electronic Resource]* **6**, 140, doi:http://dx.doi.org/10.1186/1756-3305-6-140 (2013).

[5] Miao, Q. *et al.* Seroprevalence of Toxoplasma gondii in horses and donkeys in Yunnan Province, Southwestern China. *Parasites & Vectors [Electronic Resource]* **6**, 168, doi:http://dx.doi.org/10.1186/1756-3305-6-168 (2013).
