# Supplementary material for: Seroprevalence of Toxoplasma gondii in pregnant women and livestock in the mainland of China: a systematic review and hierarchical meta-analysis
Source: Sci Rep. 2018 Apr 18;8:6218. doi: 10.1038/s41598-018-24361-8 (PMC5906581; doi:10.1038/s41598-018-24361-8)
Supplement: Supplementary file 2 — Supplementary S2 [file 41598_2018_24361_MOESM2_ESM.pdf]

Seroprevalence of *Toxoplasma gondii* in pregnant women and livestock in the mainland of China: a systematic review and hierarchical meta-analysis

Huifang Deng[1], Brecht Devleesschauwer[2], Mingyuan Liu[3], Jianhua Li[3], Yongning Wu[4], Joke van der Giessen[1], and Marieke Opsteegh[1,*]

[1] Centre for Infectious Disease Control - Zoonoses and Environmental Microbiology, National Institute for Public Health and the Environment, 3720 BA, Bilthoven, The Netherlands

[2] Department of Public Health and Surveillance, Scientific Institute of Public Health (WIV-ISP), 1050, Brussels, Belgium

[3] Institute of Zoonosis, Jilin University, 130062, Changchun, People's Republic of China

[4] Key Laboratory of China Food Safety Risk Assessment, National Center for Food Safety Risk Assessment, 100022, Beijing, People's Republic of China

* Corresponding author
E-mail: marieke.opsteegh@rivm.nl (MO)

JAGS model for pregnant woman

```
model {
  for (i in 1:N) {
    x[i] ~ dbin(ap[i], n[i])
    ap[i] <- se[methodtype[i]] * tp[i] + (1 - sp[methodtype[i]]) * (1 - tp[i])
    logit(tp[i]) <- tp.logit[i]
    tp.logit[i] ~ dnorm(logit.tp.reg[reg[i]], tau.within)
  }

  for (r in 1:NR) {
    logit(tp.reg[r]) <- logit.tp.reg[r]
    logit.tp.reg[r] ~ dnorm(logit.tp.global, tau.between)
  }

  logit(tp.global) <- logit.tp.global
  logit.tp.global ~ dnorm(0, 0.00001)

  tau.between   <- 1 / pow(sd.tau, 2)
  sd.tau        <- abs(z) / sqrt(gamma)
  z             ~  dnorm(0, inv.B.squared)
  inv.B.squared <- 1 / pow(1, 2)
  gamma         ~ dgamma(0.5, 0.5)

  tau.within <- 1 / pow(sd, 2)
  sd ~ dunif(0, 10)

  se[1] ~ dbeta(112, 1)T(0.1,)
  se[2] ~ dbeta(17, 1)T(0.1,)
  se[3] ~ dbeta(17, 1)T(0.1,)
  se[4] ~ dbeta(112,1)T(0.1,)
  se[5] ~ dbeta(38, 5 )T(0.1,)
  se[6] ~ dbeta(34, 1)T(0.1,)
  se[7] ~ dbeta(6.9, 0.4)T(0.5,)
  se[8] ~ dbeta(2, 2)T(0.1,)
  se[9] ~ dbeta(167, 19)T(0.1,)
  se[10] ~ dbeta(6.4, 1.1)T(0.1,)
  se[11] ~ dbeta(24, 2 )T(0.1,)

  sp[1] ~ dbeta(261, 4)T(0.1,)
  sp[2] ~ dbeta(72, 2)T(0.1,)
  sp[3] ~ dbeta(75, 1)T(0.1,)
  sp[4] ~ dbeta(266, 1)T(0.1,)
  sp[5] ~ dbeta(64, 1)T(0.1,)
  sp[6] ~ dbeta(36, 1.5)T(0.1,)
  sp[7] ~ dbeta(1.5, 1.8)T(0.5,)
  sp[8] ~ dbeta(7.9, 0.9)T(0.1,)
```

```
  sp[9] ~ dbeta(114, 4)T(0.1,)
  sp[10] ~ dbeta(20, 1.1)T(0.1,)
  sp[11] ~ dbeta(24, 2.4)T(0.1,)
}
```
